# Next Generation Sequencing Identifies Five Major Classes of Potentially Therapeutic Enzymes Secreted by* Lucilia sericata* Medical Maggots

**DOI:** 10.1155/2016/8285428

**Published:** 2016-03-28

**Authors:** Zdeněk Franta, Heiko Vogel, Rüdiger Lehmann, Oliver Rupp, Alexander Goesmann, Andreas Vilcinskas

**Affiliations:** ^1^Department of Bioresources, Fraunhofer Institute for Molecular Biology and Applied Ecology, Winchesterstraße 2, 35394 Giessen, Germany; ^2^Department of Entomology, Max Planck Institute for Chemical Ecology, Hans-Knöll-Straße 8, 07745 Jena, Germany; ^3^Justus-Liebig-University of Giessen, Bioinformatics and System Biology, Heinrich-Buff-Ring 58, 35392 Giessen, Germany; ^4^Justus-Liebig-University of Giessen, Institute for Insect Biotechnology, Heinrich-Buff-Ring 26-32, 35392 Giessen, Germany

## Abstract

*Lucilia sericata* larvae are used as an alternative treatment for recalcitrant and chronic wounds. Their excretions/secretions contain molecules that facilitate tissue debridement, disinfect, or accelerate wound healing and have therefore been recognized as a potential source of novel therapeutic compounds. Among the substances present in excretions/secretions various peptidase activities promoting the wound healing processes have been detected but the peptidases responsible for these activities remain mostly unidentified. To explore these enzymes we applied next generation sequencing to analyze the transcriptomes of different maggot tissues (salivary glands, gut, and crop) associated with the production of excretions/secretions and/or with digestion as well as the rest of the larval body. As a result we obtained more than 123.8 million paired-end reads, which were assembled* de novo* using Trinity and Oases assemblers, yielding 41,421 contigs with an N50 contig length of 2.22 kb and a total length of 67.79 Mb. BLASTp analysis against the MEROPS database identified 1729 contigs in 577 clusters encoding five peptidase classes (serine, cysteine, aspartic, threonine, and metallopeptidases), which were assigned to 26 clans, 48 families, and 185 peptidase species. The individual enzymes were differentially expressed among maggot tissues and included peptidase activities related to the therapeutic effects of maggot excretions/secretions.

## 1. Introduction

The maggots of certain flies have been used as traditional medicines for centuries [1] but modern maggot debridement therapy (MDT) was established approximately 100 years ago. MDT was then widely used for the treatment of chronic wounds until the mid-1940s, since when the technique has been supplanted by antibiotics and improved wound care [2]. MDT, using exclusively* Lucilia sericata* maggots, has recently undergone a renaissance, and medical maggots are now approved as an alternative approach for the treatment of many types of chronic and necrotic wounds, including diabetic ulcers [3–5], postsurgical wounds [6], and burns [7, 8]. Maggots applied to hard to heal wounds debride the necrotic tissue, disinfect the wound, and stimulate the healing process [9]. The beneficial effect of MDT cannot be attributed to the single molecule but rather to the synergistic action of various bioactive substances, including large variety of proteolytic enzymes, which are present in maggots excretions/secretions products (MEP) [10].

Debridement, the removal of necrotic tissue and wound slough, is a well-documented effect of MDT [11–13]. The maggots perform physical debridement with their mandibles, but chemical debridement with enzymes is the most important component. They do so by releasing their digestive enzymes into the wound, which liquefy necrotic and infected tissues, before it is consumed back. Chambers et al. identified three classes of proteolytic enzymes (aspartic, serine, and metallopeptidases) from MEP and proposed that mainly serine peptidases are responsible for the superficial debridement activity of maggots [14]. Only two such peptidases (serine peptidases) have been identified and characterized thus far. Chymotrypsin 1 was identified from MEP and produced in the recombinant form [15]. Recombinant enzyme was shown to degrade the eschar from venous leg ulcers* in vitro* [15] and to be unaffected by two endogenous inhibitors, *α*1-antichymotrypsin and *α*1-antitrypsin from wound eschar [16]. We recently produced and characterized Jonah-like chymotrypsin, which digested three specific extracellular matrix proteins (laminin, fibronectin, and collagen IV)* in vitro* and proposed its function in wound debridement [17].

The natural habitat of* L. sericata* larvae is rotting organic matter such as cadavers and excrement, but this ecological niche also favors many microorganisms so the larvae must have adequate defenses against infection. The maggots therefore protect themselves by producing many antimicrobial substances [18–22] and by digesting microbes, which are thus eliminated in the larval gut [23, 24]. Interestingly, MEPs also show activity against relevant human pathogens including antibiotic-resistant bacterial strains [25–27] and biofilms [28–31]. Recently, two molecules with antibiofilm activity have been identified from MEP. Affinity purified DNase disrupted* Pseudomonas aeruginosa* biofilm [32] and recombinant chymotrypsin I was active against* Staphylococcus epidermidis* and* S. aureus* biofilms [33].

Surprisingly, medical maggots also directly promote wound healing [10]. MEPs stimulate fibroblast migration [34, 35] and proliferation [36] and increase angiogenesis [37, 38]. MEPs also influence the activation of the human complement system [39], reduce proinflammatory responses [40–43], and induce fibrinolysis [44]. Recently we discovered that MEPs contain peptidases that influence blood coagulation as part of the wound healing process [45] and this activity was attributed to Jonah-like serine peptidase. Recombinant enzyme was shown to reduce the clotting time of human plasma by substituting for the intrinsic clotting factors kallikrein, factor XI, and factor XII, respectively [17].

However, although several molecules have been identified from MEP, it is still recognized as a largely unexplored source of compounds with therapeutic potential. The future studies shall focus on identification, isolation, and/or production of effector molecules and testing of their therapeutic potential. Here, we analyzed the transcriptome of different larval tissues to systematically identify MEP peptidases. It is not clear whether MEP components are exclusively produced by salivary glands or also by other tissues, so we dissected three individual tissues (gut, crop, and salivary glands) as well as the remaining larval biomass to generate tissue-specific sequence data. The extracted mRNA was sequenced using the Illumina HiSeq2000 Genome Analyzer platform and paired-end read technology. After preprocessing, 123,856,654 paired reads remained in the panel of libraries. These were processed further to yield a final assembly of 41,421 contigs in 17,479 clusters, resulting in the identification of 1729 contigs in 577 clusters encoding five different functional classes of proteolytic enzymes.

## 2. Materials and Methods

### 2.1. Preparation of Biological Material

First-instar* L. sericata* maggots were obtained from BioMonde GmbH (Barsbüttel, Germany) and were cultured under sterile conditions on Columbia agar plates with “sheep blood +” (Oxoid Deutschland GmbH, Wesel, Germany) at 28°C for 48 h in the dark. The larvae were cleaned and then infected with a mixture of* Pseudomonas aeruginosa* (DSM 50071) and* Staphylococcus aureus* (DSM 2569) as previously described [21]. The larval gut, salivary glands, and crop were dissected under a binocular microscope 8 h after infection. Dissected tissues and the remaining larval body for Illumina sequencing were frozen in liquid nitrogen and stored at –80°C. Samples for qRT-PCR analysis were processed immediately as described below.

### 2.2. RNA Isolation and Illumina Sequencing

Total RNA was extracted from individual tissues and the rest of the larval body using the innuPREP RNA Mini Isolation Kit (Analytik Jena, Jena, Germany) following the manufacturer's instructions. Additional RNA purification, quantification, and quality control were carried out as previously described [46]. An additional Turbo DNase treatment (Thermo Fisher Scientific, Waltham, MA, USA) was applied before the second purification step to eliminate contaminating DNA. The DNase was removed and the RNA purified using the RNeasy MinElute Clean up Kit (Qiagen, Hilden, Germany) following the manufacturer's protocol. RNA was eluted in 20 *μ*L Ambion RNA Storage Solution (Thermo Fisher Scientific) and poly(A)^+^ mRNA was prepared using the Ambion MicroPoly(A) Purist Kit according to the manufacturer's instructions (Thermo Fisher Scientific). The integrity and quantity of the mRNA was confirmed using an Agilent 2100 Bioanalyser and RNA Nano chips (Agilent Technologies, Santa Clara, CA, USA).

Transcriptome sequencing was carried out on an Illumina HiSeq2000 Genome Analyzer platform using paired-end (2 × 100 bp) read technology for the larval tissues, with RNA fragmented to an average length of 150 nucleotides. Sequencing was carried out by Eurofins MWG Operon (Ebersberg, Germany) and resulted in totals of 29, 33, 26, and 34 million reads for the rest of body, gut, crop, and salivary glands, respectively.

### 2.3. Read Preprocessing

The quality of the reads was checked using the FastQC toolkit [47]. Trimmomatic [48] was used to clip adapters and trim low-quality regions (parameter ILLUMINACLIP:TruSeq3-SE-2:2:30:10 SLIDINGWINDOW:5:20 HEADCROP:15 MINLEN:50). The reads from all libraries were then pooled for assembly and digitally normalized to achieve 100-fold coverage using the Trinity “*in silico* normalization” tool [49].

### 2.4. Assembly

The reads from all libraries were assembled* de novo* in two steps. One assembly was computed using the Trinity assembler [49] followed by 28 individual Velvet/Oases assemblies [50] with k-mer parameters ranging from 21 to 75. In the second step, the resulting transcript sequences were combined and high-quality sequences were extracted using the EvidentialGene pipeline [51]. Potential isoforms were detected by clustering the protein sequences from the EvidentialGene pipeline using CD-HIT [52] with 90% identity.

### 2.5. Quality Assessment

The CEGMA method [53] was used to assess the completeness of the transcriptome. The detection step of the CEGMA pipeline was replaced by a BLASTp search [54] against the CEGMA EuKaryotic Orthologous Groups (KOG) sequences, because protein sequences had already been identified in the previous step. The completeness of individual sequences was estimated by computing the “ortholog hit ratio” [55] against the* D. melanogaster* protein sequences.

### 2.6. Functional Annotation and Peptidase Identification

Putative transposable elements were identified using TransposonPSI [56]. Furthermore, all sequences with HMMER 3.0 [57] hits against Pfam domains [58] as previously described [59] were marked as potential transposable elements. All sequences in clusters with at least one putative transposable element were annotated as transposable elements. All sequences were uploaded to the SAMS web server [60] and automatically annotated using BLAST [54] and HMMER [57] searches against different databases. Next all the peptidases were identified using the EC numbers [61] from the automatic annotation of the transcriptome data and further classified using MEROPS database [62].

### 2.7. Mapping and Digital Gene Expression Analysis

Digital gene expression analysis was carried out by using QSeq Software (DNAStar Inc.) to remap the Illumina reads onto the reference backbone and then counting the sequences to estimate expression levels. For read mapping, we used the following parameters: n-mer length = 40; read assignment quality options required at least 40 bases (the amount of mappable sequence as a criterion for inclusion) and at least 90% of bases matching (minimum similarity fraction, defining the degree of preciseness) within each read to be assigned to a specific contig; maximum number of hits for a read (reads matching a greater number of distinct places than this number are excluded) = 10; n-mer repeat settings were automatically determined and other settings were not changed. Biases in the sequence datasets and different transcript sizes were corrected using the RPKM algorithm (reads per kilobase of transcript per million mapped reads) to obtain correct estimates for relative expression levels. For the selected protease groups, gene expression (log2 transformed RPKM values) was visualized as heat maps using custom scripts and matplotlib [63] to generate a 2D plotting library using the Jet Colormap [64].

### 2.8. Quantitative Reverse Transcription Real-Time PCR Reaction (qRT-PCR)

A subset of differentially expressed peptidases from each peptidase clan was validated by qRT-PCR. Total RNA isolation, cDNA synthesis, primer design, and qRT-PCR experiments were performed as described previously [65]. Data were analyzed in Rest 2009 (http://www.gene-quantification.de/rest-2009.html) using the ΔΔCq method [66]. Relative mRNA values of individual genes were adjusted to the sample with the highest value and normalized using the* 60S acidic ribosomal protein P0* (RPLPO) and* 40S ribosomal protein S3* (RPS3) genes.

## 3. Results and Discussion

### 3.1. Preprocessing and Assembly of Sequence Reads

After preprocessing, 123,856,654 paired reads remained in the four tissue-specific libraries (Table 1). The “*in silico* normalization” of the pooled reads reduced the total number to 16,236,645. The normalized reads were assembled using the Trinity assembler and 28 individual Velvet/Oases assemblies, producing a total of 1,794,145 contigs (Additional file 1; see Supplementary Material available online at http://dx.doi.org/10.1155/2016/8285428). Filtering and clustering of the contigs using the EvidentialGene pipeline and CD-HIT produced a final set of 41,421 contigs in 17,479 clusters. The final assembly contained 41,421 contigs covering a total of 67.79 Mb, a mean contig length of 1.64 kb, and a N50 contig length of 2.22 kb.

### 3.2. Assembly Quality Control

We found that 80–89% of the nonnormalized reads could be mapped to the final assembly (84.61% body, 80.30% crop, 88.92% gland, and 87.95% gut). CEGMA identified 235 of the 248 (94.76%) core genes with an ortholog ratio of 2.79. We then mapped 26,950* Drosophila melanogaster* protein sequences to the final assembly, and 9961 (36.96%) could be aligned with a mean ortholog hit ratio of 0.71. The mean ortholog number (number of contigs mapping to the same* D. melanogaster* protein sequence) was 3.5.

### 3.3. Annotation

**Figure 1:**
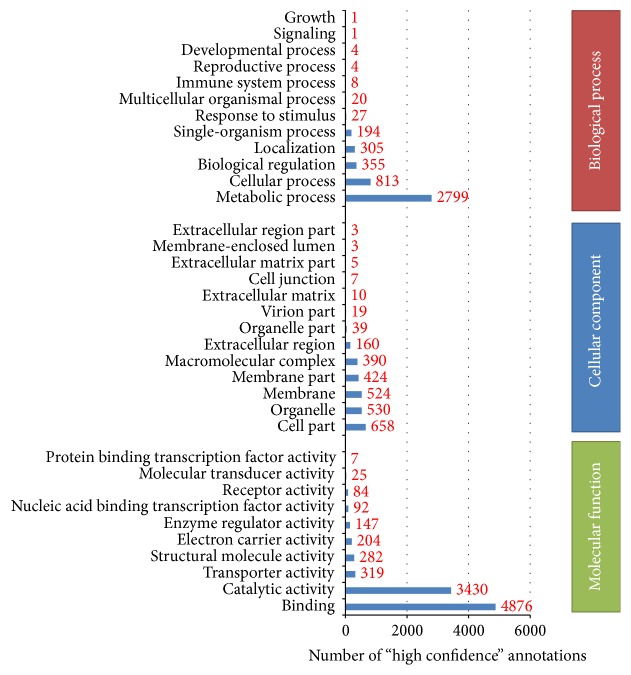
Gene ontology analysis of the* L. sericata* transcriptome. All identified contigs (41,421) were categorized using three GO terms: (a) biological process; (b) cellular component; (c) molecular function.

TransposonPSI and HMMER searches identified 470 putative transposable elements in 288 clusters. The automatic functional annotation pipeline involving BLASTp searches against different databases revealed 17,864 (43.12%) “high confidence” annotations and 15,155 (36.58%) “hypothetical proteins.” Gene ontology (GO) analysis was used to explore the functional characteristics of all contigs and assign them to three independent categories: biological processes, molecular function, and cellular components (Figure 1). In addition, a BLASTp search against the MEROPS database v9.12 [67] identified 1729 contigs in 577 clusters as peptidases. The identified peptidases represented ~4% of the total number of contigs. This result correlates with data from other organisms where peptidases represent more than 2% of all genes [68].

### 3.4. Peptidases

**Figure 2:**
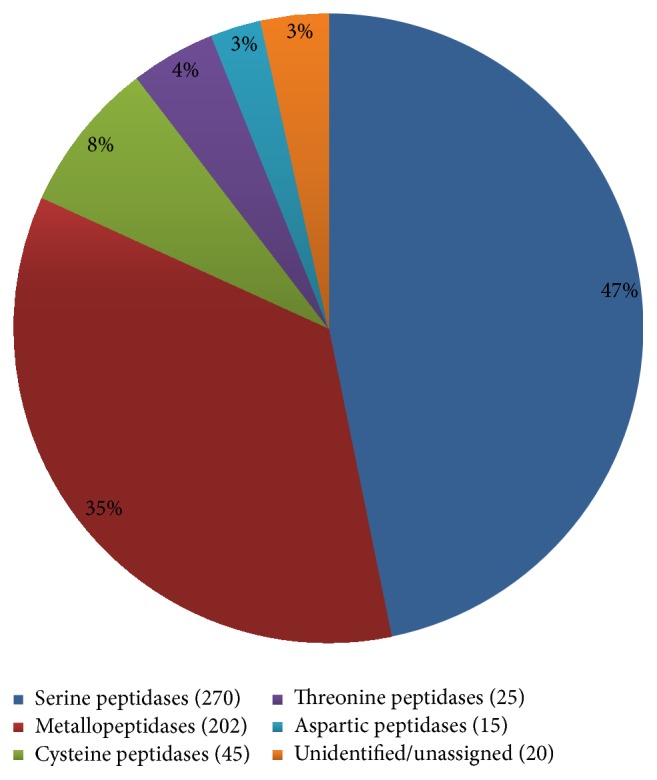
Proportional representation of each peptidase class in the* L. sericata* transcriptome. Among 577 clusters identified as peptidases, 557 clusters were assigned to 5 peptidase classes (serine peptidases (270), metallopeptidases (202), cysteine peptidases (45), threonine peptidases (25), and aspartic peptidases (15)) and 20 clusters remained unassigned.

Peptidases are proteolytic enzymes that hydrolyze peptide bonds and they are found ubiquitously in all biological systems from viruses to vertebrates. Based on the key amino acid residues responsible for proteolytic activity, six different peptidase classes are recognized (aspartic, cysteine, serine, glutamic, threonine, and metallopeptidases) as well as further unclassified peptidases [69]. From the 1729* L. sericata* contigs (in 577 clusters) identified as peptidases, 1655 contigs (in 557 clusters) were assigned to one of five peptidase classes (aspartic, cysteine, serine, threonine, and metallopeptidases) whereas 74 contigs (in 20 clusters) remained unassigned. As summarized in Figure 2, serine peptidases were the most prominent class (837 contigs in 270 clusters) followed by metallopeptidases (565 contigs in 202 clusters), cysteine peptidases (145 contigs in 45 clusters), threonine peptidases (51 contigs in 25 clusters), and aspartic peptidases (57 contigs in 15 clusters). The MEROPS database was used to subdivide the identified enzymes further into clans (peptidases with evolutionarily conserved tertiary structures, orders of catalytic residues, and common sequence motifs around the catalytic site), families (peptidases with similar amino acid sequences), and species (peptidases with similar properties and a unique MEROPS identity) [70, 71]. Accordingly we identified 26 clans containing 48 families and 185 peptidase species (Table 2). We found that almost half of the identified clusters represented serine peptidases in clan PA and family S1.

**Figure 3:**
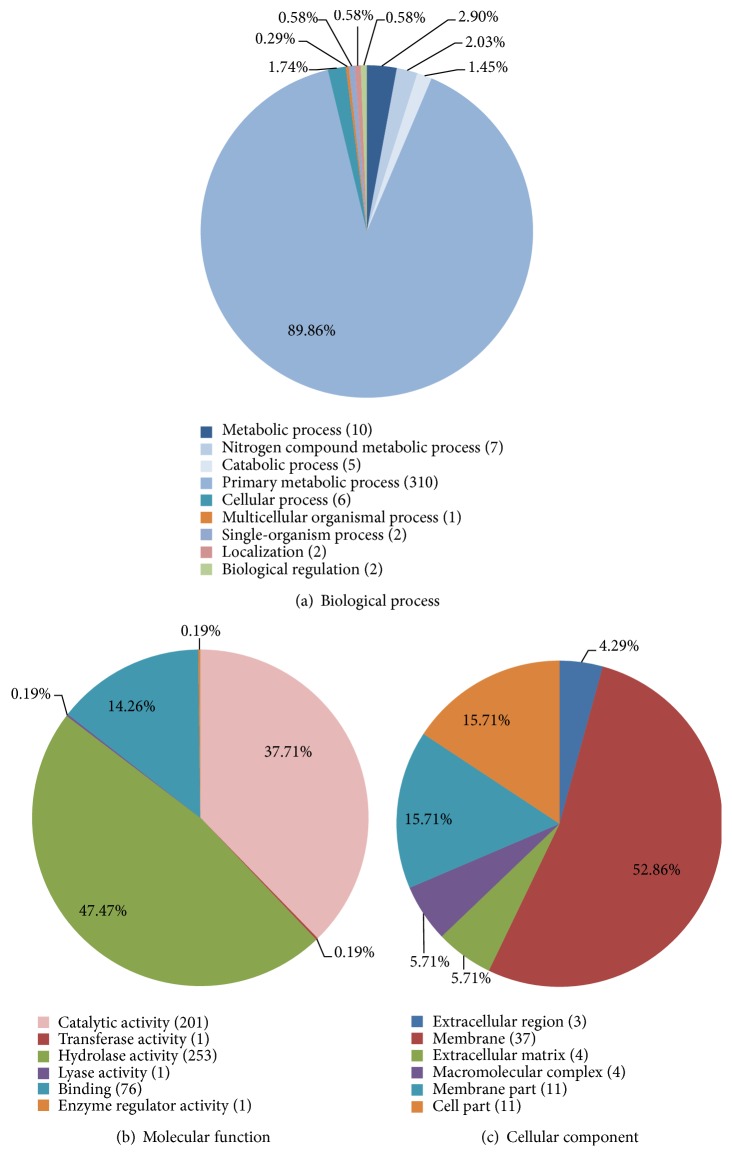
Gene ontology analysis of* L. sericata* peptidases. All identified peptidase clusters (577 in total) were categorized using three GO terms: (a) biological process, (b) molecular function, and (c) cellular component. Analysis of (a) biological process and (b) molecular function was performed at level three, whereas analysis of (c) cellular component was performed at level two.

GO analysis was then carried out to assign functional categories to each of the identified peptidase clusters. We were able to assign 534 of 577 clusters to three different categories: biological process (345 clusters), molecular function (533 clusters), and cellular component (70 clusters) (Figure 3). We found that most of the peptidases (310 clusters) are involved in the biological process (level 3) category of “primary metabolic process” (Figure 3(a)). The molecular function (level 3) of most peptidases was either catalytic activity (201) or hydrolase activity (253) (Figure 3(b)) as expected given the molecular role of peptidases. Interestingly, only 70 clusters were assigned a cellular component function (Figure 3(c)).

### 3.5. Aspartic Peptidases

Aspartic peptidases contain an aspartic acid residue at the active site [72]. An aspartic peptidase activity was previously identified in maggot MEPs using class-specific inhibitors [14] and the corresponding gene was shown to be strongly upregulated in* L. sericata* larvae following an immune challenge [18]. We identified 57 contigs in 15 clusters representing aspartic peptidases, and these were further assigned to two clans, three families, and seven peptidase species (Table 3) with different tissue-specific expression profiles (Additional file 2).

#### 3.5.1. Family A1

**Figure 4:**
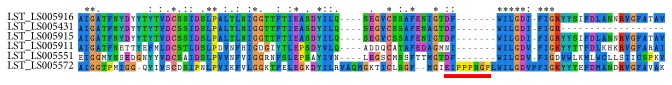
Partial amino acid sequence alignment of A1 peptidases found in the* L. sericata* transcriptome. Amino acid sequences of A1 aspartic peptidases were aligned using MAFFT [199]. Only one cluster (LST_LS005572) contained a polyproline loop (underlined). Asterisk (*∗*) indicates positions which have a single, fully conserved residue. Colon (:) indicates conservation between groups of strongly similar properties, scoring > 0.5 in the Gonnet PAM 250 matrix. Period (.) indicates conservation between groups of weakly similar properties, scoring =< 0.5 in the Gonnet PAM 250 matrix.

Preprocathepsin D-like peptidases are the largest group of aspartic peptidases. The majority of clusters included a signal peptide, propeptide, and mature enzyme containing all of the conserved catalytic and substrate-binding residues found in human lysosomal cathepsin D [69]. With the exception of cluster LST_LS5572 and two incomplete clusters (LST_LS009595 and LST_LS016491), all clusters lacked the polyproline loop (DxPxPx(G/A)P) (Figure 4). The absence of this loop is a characteristic of pepsin and digestive cathepsin D peptidases in the Brachyceran infraorder Muscomorpha and may be associated with the extracellular role of these enzymes [73]. The aspartic peptidase gene previously identified in challenged* L. sericata* larvae [GenBank: FG360526] is homologous to cluster LST_LS005916, which was predominantly expressed in the larval gut. Aspartic peptidases can kill bacteria* in vitro* in an acidic medium [74] and may also kill bacteria in the* Musca domestica* larval midgut (Espinoza-Fuentes, Terra 1987). Based on its localization in the gut and induction by an immune challenge, we propose a similar role for this* L. sericata* aspartic peptidase. However, heat map analysis (Additional file 2) revealed that the majority of A1 family aspartic peptidases are predominantly expressed in the larval gut, suggesting a role in digestion and/or the elimination of bacteria.

#### 3.5.2. Family A22

This family of intramembrane peptidases comprises two subfamilies. The A22a subfamily is typified by presenilin, an enzyme that plays central role in intramembrane proteolysis [75] and the pathogenesis of Alzheimer's disease [76]. The A22b subfamily is typified by impas 1 peptidase, which is responsible for the degradation of liberated signal peptides and may play an essential role in the development of* D. melanogaster* larvae [77]. We identified four clusters assigned to three peptidase species encoding members of the A22b subfamily, and two of them (LST_LS005714 and LST_LS015224) were strongly upregulated in the salivary glands (Additional file 2) as previously reported in* D. melanogaster* [77]. We therefore propose a similar function for the* L. sericata* peptidase in larval development.

#### 3.5.3. Family A28

Only one contig in one cluster was identified assigned to family A28. A homologous skin aspartic peptidase (SASPase) was recently identified in human skin [78] although its biological role remains unclear.

### 3.6. Cysteine Peptidases

Cysteine peptidases are ubiquitous and mediate diverse biological processes, including immune responses [79], extracellular matrix remodeling [80], and development and apoptosis [81]. The deregulation of cysteine peptidases is associated with human diseases such as cancer and atherosclerosis [82]. There are 65 cysteine peptidase families listed in MEROPS database v9.12 [62], 56 of which are assigned to 10 annotated clans, whereas 9 families remain unassigned. Our* L. sericata* transcriptome contained 145 contigs in 45 clusters belonging to 6 clans, 11 families, and 23 peptidase species (Table 4).

#### 3.6.1. Family C1

The biggest family of cysteine peptidases in the* L. sericata* transcriptome is clan CA family C1, and its members were moderately abundant in all tissues (Additional file 3). Of 16 clusters, 12 were assigned to 7 peptide species including several with known roles. These include insect 26/29 kDA peptidase, which plays a role in immunity [83, 84], vitellogenic cathepsin B, which degrades vitellogenin [85], and bleomycin hydrolase, whose natural function remains unknown although it can inactivate bleomycin [69]. Heat map analysis (Additional file 4) revealed only three papain homologs present mostly in the larval gut (LST_LS004517, LST_LS006643, and LST_LS006644), and two of them (LST_LS004517 and LST_LS006644) are identical to previously identified partial sequences of* L. sericata* cysteine peptidases [GenBank: FG360492, FG360504] that are upregulated in response to an immune challenge [18]. Although, papain-type cysteine peptidases were previously shown to play important digestive role in ticks [86], hemipterans [87], and beetles [88], their digestive role in dipteran species remains unclear [89]. Based on the strict localization in the* L. sericata* gut and induction by an immune challenge, we speculate that these enzymes are probably required for the elimination of bacteria in the larval gut [23] rather than the digestion of food, but additional experiments are needed to confirm their specific function.

#### 3.6.2. Family C13

The C13 family of cysteine peptidases comprises two types of enzymes. The first is the asparaginyl endopeptidases, which were originally found in legumes [90] and later in schistosomes [91], mammals [92], and recently also arthropods [93]. These are acidic lysosomal enzymes that favor asparagine at the P1 position [94] and whose roles include antigen presentation [95], enzyme transactivation [96], and blood meal digestion [97]. The second is the glycosylphosphatidylinositol (GPI):protein transamidases, which are required for the removal of C-terminal peptides and the attachment of GPI anchors [98]. We identified two clusters encoding GPI:protein transamidases and two clusters remained unidentified.

#### 3.6.3. Family C14

**Figure 5:**
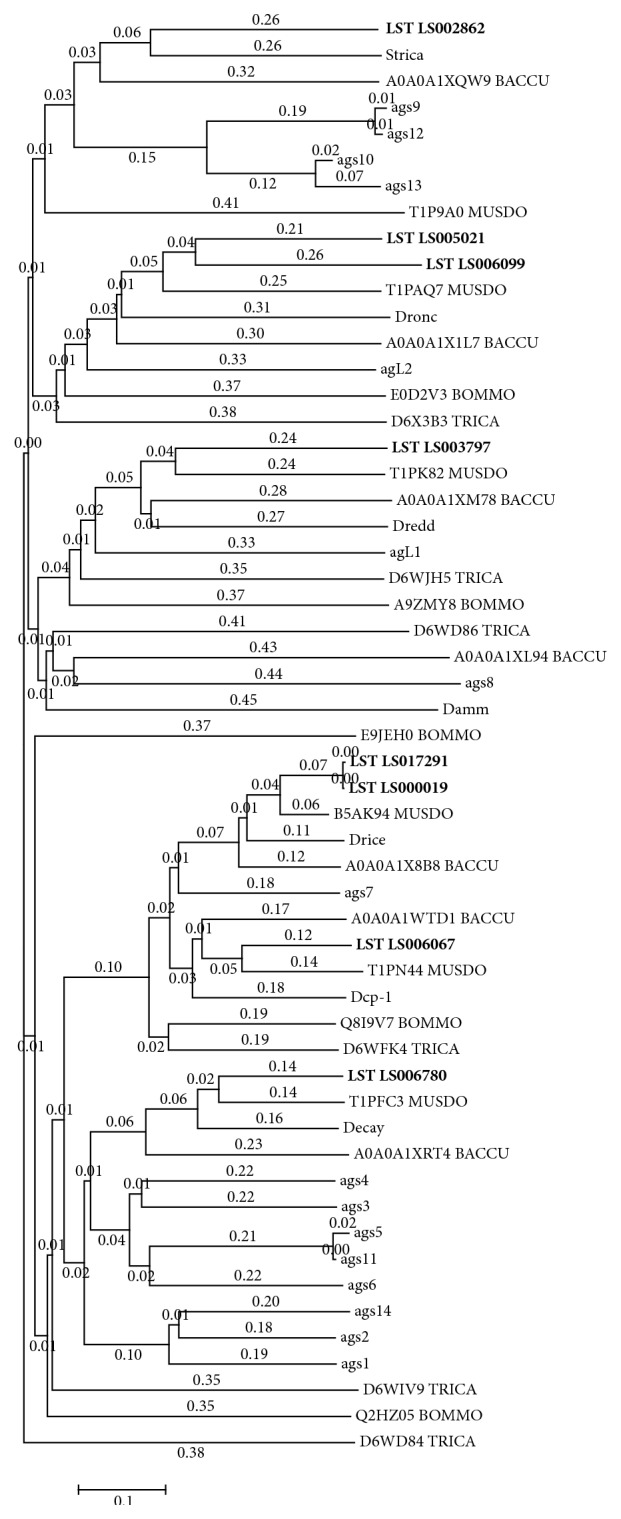
Phylogenetic analysis of* L. sericata* caspases. The phylogenetic analysis of eight identified caspases in the transcriptome of* L. sericata*. For the analysis the sequences were compared with* Bombyx mori* (BOMMO), UniProtKB [E0D2V3; E9JEH0; E0D2V3; E9JEH0; Q2HZ05; Q8I9V7],* Tribolium castaneum* (TRICA), UniProtKB [D6WD84; D6WD86; D6WFK4; D6WIV9; D6WJH5; D6X3B3],* Musca domestica* (MUSDO), UniProtKB [T1P9A0; T1PAQ7; T1PFC3; T1PK82; T1PN44; B5AK94],* Bactrocera cucurbitae* (BACCU), UniProtKB [A0A0A1WTD1; A0A0A1X1L7; A0A0A1X8B8; A0A0A1XL94; A0A0A1XM78; A0A0A1XQW9; A0A0A1XRT4],* D. melanogaster* (Strica, UniProtKB [Q7KHK9]; Dronc, UniProtKB [Q9XYF4]; Dredd, UniProtKB [Q8IRY7]; Damm, UniProtKB [O44252]; Drice, UniProtKB [O01382]; Dcp-1, UniProtKB [O02002]; Decay, UniProtKB [Q9VET9]), and* Anopheles gambiae* (agL1, agL2, ags1–ags14), UniProtKB [Q5TMK1; Q7Q2Q0; Q5TMS0; Q7PZJ9; Q7PZK0; Q7PV92; Q7Q802; Q7Q803; Q7Q4X6; Q7PWK2; Q7QGM9; Q7PSJ2; Q7Q801; Q7QGN0; Q7QGM8; Q7QKT2]. For the alignment MAFFT [199] was used.

Caspases are intracellular endopeptidases that are highly specific for the cleavage of aspartyl bonds. With the exception of caspase 1, which is responsible for the production of interleukin-1*β* in monocytes [69], most caspases regulate apoptosis by taking part in a protease cascade [99]. The* D. melanogaster* genome encodes seven caspases. Dronc (*Drosophila* Nedd2-like caspase), Dredd (death related ced-3/Nedd2/like), and Strica (serine/threonine rich caspase) possess long N-terminal domains and function as upstream or initiator enzymes, whereas Drice (Drosophila ICE), Dcp-1 (death caspase-1), and Decay (death executioner caspase related to Apopain/Yama) are downstream or effector caspases [100, 101]. Damm (death-associated molecule related to Mch2) caspase shares the features of both groups but its biological role is not fully understood [69]. The* L. sericata* transcriptome database contained eight clusters in seven peptidase species representing caspases, with different tissue-specific expression profiles (Additional file 4). Phylogenetic analysis (Figure 5) revealed one* L. sericata* homolog each for the effector caspases Dcp-1 and Decay, two homologs for Drice, one homolog each for the initiator caspases Dredd and Strica, and two homologs for Dronc. We did not find a sequence representing the* D. melanogaster* Damm caspase.

#### 3.6.4. Other Cysteine Peptidase Families

Several cysteine peptidase families were more or less equally distributed among the* L. sericata* tissues we tested, and these are probably required for essential cellular functions. The C2 family of calcium-dependent peptidases (calpains) is formed of ubiquitous, intracellular, neutral peptidases, associated with diverse biological functions ranging from signal transduction to apoptosis [102]. Ubiquitinyl hydrolases (family C12) are intracellular enzymes that remove ubiquitin from ubiquitinylated proteins and peptides [69]. Members of family C15 are ubiquitous, intracellular peptidases that remove pyroglutamate from the N-terminus of peptides and hydrolyze biologically active peptides such as neurotensin and gonadotropin [103]. Gamma-glutamyl hydrolases (family C26) are primarily lysosomal enzymes, which are widely distributed in nature and probably required for the turnover of cellular folates [69]. Hedgehog proteins (family C46) are self-splicing, two-domain signaling proteins originally discovered in* D. melanogaster* [104]. They are found in most metazoan species and play multiple roles in pattern formation during development [105]. Members of family C54, which was first discovered in the budding yeast* Saccharomyces cerevisiae*, are necessary for autophagy [106]. Otubains (family C65) are isopeptidases involved in the removal of ubiquitin from polyubiquitin [107]. These enzymes share no homology to other deubiquitinylating enzymes but belong to the ovarian tumor family (OTU) and possess a cysteine peptidase domain [108].

### 3.7. Metallopeptidases

The metallopeptidases are a ubiquitous and highly diverse group of enzymes containing both endopeptidases and exopeptidases. MEROPS database v9.12 [62] lists more than 15 clans and 71 families involved in diverse biological processes such as digestion, wound healing, reproduction, and host-pathogen interactions. Although these enzymes vary widely at the sequence, structural, and even functional levels, all members require a metal ion for catalytic activity [69]. More than 30% of all clusters in our* L. sericata* transcriptome database (202 clusters) were found to encode metallopeptidases, which were further assigned to 9 clans, 20 families, and 53 peptidase species (Table 5). The metallopeptidases are therefore the second largest group of peptidases in the* L. sericata* transcriptome and the most diverse in terms of the number of families. Although the variability and abundance of metallopeptidases in* L. sericata* indicate their importance, their roles are not well understood and few studies have addressed specific biological activities. A metallopeptidase with exopeptidase characteristics and a pH optimum of 8 was detected in* L. sericata* MEPs using FITC-casein as a substrate [14] and a partial sequence encoding a clan MC; family M14 metallopeptidase [GenBank: FG360509] was identified among the upregulated genes in immune challenged larvae [18].

#### 3.7.1. Family M1

Family M1 is one of 16 peptidase families found in the genomes of all forms of cellular life [109]. This family mostly comprises membrane-bound or cytosolic exopeptidases that remove the N-terminus of their substrates. However the specificity of the S1 subsite varies considerably, which allows this family to be involved in many different biological processes [110]. Insect M1 peptidases are mainly expressed in the gut, where they play important intermediate roles in protein digestion [111] as well as host-pathogen interactions. Membrane-bound aminopeptidases in the gut are receptors for* Bacillus thuringiensis* toxins in several insect species [112–114]. Aminopeptidases have also been detected in other insect tissues, such as the fat body [115], salivary glands [116], and Malpighian tubes [116]. Although their interactions with* B. thuringiensis* toxins have been confirmed, their endogenous role is unclear [116]. Aminopeptidase N in the hemocoel plays an important role in the postembryonic development of the pest moth* Achaea janata* [117]. We identified 33 clusters encoding 8 peptidase species (Table 5) and 6 of them are predominantly expressed in the larval gut (Additional file 4).

#### 3.7.2. Family M2

Family M2 contains angiotensin converting enzyme (ACE), the dipeptidyl peptidase that removes dipeptides from the C-terminus of angiotensin. ACE was originally identified in mammals, where it regulates vascular homeostasis [69]. The first insect ACE was found in* M. domestica* [118] and several ACE paralogs have been identified in every insect genome investigated thus far [119]. Insect ACE cleaves peptides with roles in development [119, 120], reproduction [121], and immunity [122, 123]. Recently, ACE was shown to be involved in aphid-plant interactions by modulating the feeding behavior and survival of aphids on plants [124]. Six ACE paralogs were identified in the* D. melanogaster* genome, but only Ance and Acer are active enzymes [125]. These enzymes have distinct tissue localization and substrate profiles, but their exact role is unclear. Ance is expressed mostly in the gut and around the reproductive organs, thus suggesting a role processing peptides in gut muscle cells [126] and during spermatogenesis [121]. In contrast, Acer was exclusively found in developing heart cells [127]. We identified 26 clusters belonging to the M2 family, 16 of which were assigned to the Ance peptidyl-dipeptidase species (Table 5) and were predominantly expressed in the larval gut (Additional file 4). Based on this localization, we speculate that* L. sericata* Ance plays a similar role to its* D. melanogaster* ortholog. Interestingly,* D. melanogaster* Ance was shown to hydrolyze the two important bioactive peptides angiotensin I and bradykinin [119], which are the major substrates of mammalian ACE. It would be interesting to see whether* L. sericata* Ance can also cleave these substrates, which would suggest a potential endogenous role in hormonal signaling.

#### 3.7.3. Family M3

The* L. sericata* transcriptome was shown to contain mitochondrial intermediate peptidase and thimet oligopeptidase, which were expressed similarly in all the tissues we sampled (Additional file 4). Both enzymes are intracellular endopeptidases. Mitochondrial intermediate peptidase processes mitochondrial protein precursors during their import into the mitochondria [128], whereas thimet oligopeptidase degrades small peptides (5–53 residues) with broad specificity and plays an important role in antigen presentation [129].

#### 3.7.4. Family M8

Two* L. sericata* clusters belong to family M8, which is typified by leishmanolysin, an important virulence factor found in leishmania parasites [130]. Leishmanolysin is a membrane-bound peptidase which degrades extracellular matrix proteins, thus enabling parasite migration [131]. Furthermore, a* D. melanogaster* M8 metallopeptidase was found to be involved in cell migration during embryogenesis and coordinated mitotic progression [132].

#### 3.7.5. Family M10

Family M10 comprises secreted matrix metalloendopeptidases (MMPs) that are synthesized as inactive proenzymes and become functional in the extracellular environment. MMPs are strongly conserved and have been identified in plants [133], cnidarians [134], nematodes [135], insects (Vilcinskas and Wedde 2002), and humans [136]. As their name indicates, MMPs play important roles in extracellular matrix remodeling and turnover. Aberrant MMP activity is associated with many forms of cancer, making them medically relevant [137]. Most MMPs are oncogenic, that is, higher activity promotes cancer, but some (including MMP3 and MMP8) have the opposite effect [138]. It is difficult to determine their precise individual roles because there are 24 human MMPs with overlapping expression profiles and activities, but insects could be used as a simplified model to probe their functions in more detail. Only two* D. melanogaster* MMPs have been described [139, 140], as well as three from the red flour beetle* Tribolium castaneum* [141] and one from the greater wax moth* Galleria mellonella* [142]. All insect MMPs play important physiological roles and some also promote tumor progression, suggesting they have similar functions to their human counterparts [143]. We identified only four clusters representing* L. sericata* MMPs, which were assigned to two peptidase species (Table 5). These enzymes were generally expressed at low levels but were slightly upregulated in the larval gut (Additional file 4). The role of these enzymes remains unknown and further studies are required to clarify their physiological functions and whether* L. sericata* MMPs contribute to the degradation of extracellular matrix proteins in human wounds.

#### 3.7.6. Family M12

Family M12 comprises two subfamilies, namely, subfamily M12a, which is typified by astacin, and subfamily M12b, which is typified by adamalysin. Astacin is an endopeptidase, originally identified in the crayfish* Astacus astacus*, which is probably involved in digestion [69]. Hundreds of astacins have been identified in many different species, but no examples have yet been identified in plants and fungi [144]. In addition to digestion, astacins may also play roles in embryogenesis and extracellular protein remodeling [145]. Adamalysins are membrane-bound proteins with disintegrin and metallopeptidase domains. They have a broad substrate range and are therefore involved in many important physiological processes, such as protein shedding, development, and spermatogenesis [146]. Adamalysins are also known to facilitate cell signaling and have been implicated in carcinogenesis, making them medically relevant [147]. We identified 13* L. sericata* clusters representing subfamily M12a and another 13 clusters representing subfamily M12b. Only one cluster (LST_LS007850) was mainly expressed in the larval gut, indicating a potential role in digestion, whereas the others showed diverse tissue-specific expression profiles and their roles remain unclear.

#### 3.7.7. Family M13

Neprilysin and endothelin converting enzyme (ECE) are the two best characterized members of metallopeptidase family M13 in mammals. Neprilysin is involved in biological processes such as reproduction and the modulation of neuronal activity and blood pressure, whereas ECEs are responsible for the final step in the synthesis of endothelins, which are potent vasoconstrictors [69]. Insect family M13 metallopeptidases are membrane-bound peptidases with a broad substrate range and tissue distribution [125]. The precise biological roles of these enzymes in insects are still unclear, but they are associated with immunity to bacteria, fungi, and protozoa [122, 148], metamorphosis [149], reproduction [150], and neuropeptide metabolism [151]. We identified 34 clusters coding for M13 peptidases in* L. sericata* and they were predominantly expressed in the larval body following the removal of the gut, crop, and salivary glands (Additional file 4). Among 34 clusters, 15 were further assigned to 8 peptidase families, whereas 13 remained unassigned and 6 represent nonpeptidase homologs (Table 5).

#### 3.7.8. Family M14

Most family M14 enzymes are carboxypeptidases that remove a single amino acid residue from the C-terminus of polypeptides. Carboxypeptidases are required for digestion and are widely distributed among insects [152], but they also process bioactive peptides (carboxypeptidase E) and hydrolyze bacterial cell walls (*γ*-glutamyl-(L)-meso-diaminopimelate peptidase I) [69]. Recently, a partial* L. sericata* sequence encoding an M14 metallopeptidase was found to be upregulated by an immune challenge [18]. We identified 33 clusters representing M14 family metallopeptidases that were differentially expressed among the* L. sericata* tissues we tested (Additional file 4). These clusters were assigned to 9 peptidase species, whereas 14 remained unassigned and one was shown to represent a nonpeptidase homolog (Table 5). The previously identified M14 peptidase [GenBank: FG360509] was found to be homologous to cluster LST_LS004029, which is strongly expressed in the gut. The localization of this enzyme in the gut and its induction in response to an immune challenge suggest that it contributes to the elimination of ingested bacteria as previously described for* L. sericata* larvae [23].

#### 3.7.9. Family M16

Family M16 can be divided into three subfamilies: M16A comprising oligopeptidases such as insulysin, nardilysin and pitrilysin, M16B which includes mitochondrial processing peptidase, and M16C which includes eupitrilysin [69]. We identified four M16A clusters and two peptidase species with pitrilysin-like characteristics. Pitrilysin is an endopeptidase originally found in* Escherichia coli* which is homologous to human insulin-degrading enzyme [153]. We also identified five M16B clusters and three peptidase species similar to mitochondrial processing peptidase, which cleaves the N-terminal signals of mitochondrial proteins during their import from the cytosol [69]. We also identified one M16C cluster representing one peptidase species similar to eupitrilysin.

#### 3.7.10. Family M17

Leucyl aminopeptidase (LAP) is a cocatalytic peptidase; that is, it requires two metal ions for activity, with diverse biological roles [154]. We identified four clusters and two peptidase species similar to LAP, with the strongest expression in gut tissues (Additional file 4). LAPs were previously identified in the digestive organs of blood-feeding parasites including ticks [155], schistosomes [156], and* Plasmodium* spp. [157] and were found to be involved in the final stage of hemoglobin digestion. Because hemoglobin could also represent part of the* L. sericata* diet, we can speculate that* L. sericata* LAPs similarly are required for hemoglobin digestion.

#### 3.7.11. Families M19 and M50

Families M19 and M50 each comprise strictly membrane-bound enzymes. The family M19 dipeptidases degrade extracellular glutathione or inactivated leukotriene D4, whereas the family M50 enzymes regulate gene expression by processing different transcription factors [69]. We identified one cluster coding for a family M19 nonpeptidase homolog and one representing a family M50 S2P peptidase. The latter is a strongly hydrophobic peptidase found on the endoplasmic reticulum membrane.* D. melanogaster* S2P (ds2p) is required to cleave the sterol regulatory element binding protein (SREBP) and thus helps to regulate lipid biosynthesis [158].

#### 3.7.12. Families M20 and M28

Families M20 and M28 comprise divergent cocatalytic exopeptidases. Family M20 contains only carboxypeptidases, whereas family M28 includes both carboxypeptidases and aminopeptidases. We identified one cluster in family M20, which was tentatively identified as peptidase T and one cluster tentatively identified as a homolog of* D. melanogaster* putative protein CG10062. Six unassigned clusters were also identified in family M28.

#### 3.7.13. Family M24

Members of family M24 are mostly intracellular, cocatalytic exopeptidases characterized by the so-called* pita-bread fold* [159], which have been found in every genome sequence published thus far [109]. They are involved in many fundamental biological processes, including the removal of N-terminal methionine residues from nascent polypeptides (methionyl aminopeptidase), intracellular protein turnover, and collagen metabolism (Xaa-Pro dipeptidase). They are also involved in angiogenesis and their specific inhibitors are therefore sought as potential anticancer drugs [160]. We identified nine clusters representing methionyl aminopeptidases and one Xaa-Pro dipeptidase. Clusters LST_LS003866, LST_LS017277, and LST_LS003028 were predominantly expressed in the larval gut, whereas the other M24 family metallopeptidases were expressed at similar levels in all the tissues we investigated (Additional file 4).

#### 3.7.14. Families M48 and M79

The members of families M48 and M79 are membrane-bound metallopeptidases involved in the release of tripeptides from* Saccharomyces cerevisiae α* mating factor [161] and the Ras oncoprotein [162], to facilitate membrane attachment. Both families are medically relevant because of their ability to regulate the function of Ras, which is involved in many forms of cancer. We identified two clusters in one peptidase species coding for M48 family and one cluster in one peptidase species coding for M79. All clusters were expressed at similar levels in all dissected tissues.

#### 3.7.15. Family M49

Dipeptidyl-aminopeptidase III (DPP-3) is an exopeptidase that may be involved in the metabolism of angiotensin peptide and encephalin in mammals [163]. Insect orthologs of DPP-3 were purified from the foregut membrane of the cockroach* Blaberus craniifer* [164] and from adult* D. melanogaster* [165]. Purified DPP-3 hydrolyzed an insect neuropeptide (proctocolin), suggesting a role in neuropeptide signaling activity. We identified two clusters and one peptidase species related to DPP-3 expressed at similar levels in all the* L. sericata* tissues we tested.

#### 3.7.16. Family M67

Family M67 metallopeptidases are responsible for the removal of ubiquitin from ubiquitinylated proteins prior to their degradation in the proteasome. We identified one cluster and one peptidase species representing family M67 expressed at similar levels in all the* L. sericata* tissues we tested.

### 3.8. Threonine Peptidases

Threonine peptidases were discovered in 1995 in archaean proteasomes [166]. They are N-terminal nucleophile peptidases belonging to clan PB and can be divided into three families, namely T1, T2, and T3. The T1 family comprises peptidases of the proteasome and related compound peptidases. The proteasome plays a central role in intracellular protein turnover and is a complex supramolecular complex with many subunits [167]. The T2 family comprises the aspartyl glucosylaminases, which are necessary for the degradation of asparagine-linked glycoproteins [168]. The T3 family comprises the *γ*-glutamyltransferases, which play a key role in glutathione metabolism [69]. Among 25* L. sericata* clusters identified as threonine peptidases, 21 clusters and 14 peptidase species represented family T1, whereas 4 clusters and 2 peptidase species represented family T2 (Table 6). All 25 clusters were expressed in all the* L. sericata* tissues we investigated and the expression levels were universally low (Additional file 5).

### 3.9. Serine Peptidases

Serine peptidases require a serine residue for their catalytic activity and represent one of the most abundant and functionally diverse groups of enzymes. Serine peptidases are involved in a broad range of biological processes including digestion, development, immunity, and blood coagulation [169]. MEROPS database v9.12 [62] lists 45 families in 15 clans as well as further 7 unassigned families. We found that serine peptidases are the largest group of peptidases in the* L. sericata* transcriptome. We identified more than 800 contigs in 270 clusters, which were assigned to 8 clans, 12 families, and 86 peptidase species (Table 7). These clusters showed a number of distinct tissue-specific expression profiles (Additional file 6).

#### 3.9.1. Family S1

Clan PA family S1 comprises endopeptidases containing the catalytic triad His-Asp-Ser, and this was the largest peptidase family we found in the* L. sericata* transcriptome. Most S1 peptidases possess an N-terminal signal peptide and are synthesized as propeptides that must be cleaved to generate the active form. S1 peptidases are usually soluble, secreted enzymes, but membrane-bound and inactive homologs have also been described [69]. Many S1 peptidases have been identified in insects, where their roles include digestion [111], immunity [170], wound responses [171], and development [172]. S1 serine peptidases from* L. sericata* maggots have been associated with several of the beneficial effects of MEPs including blood coagulation [45], biofilm eradication [33], and wound debridement [15]. Although serine peptidases play an important role in MDT, only a small number of complete and partial* L. sericata* sequences representing these enzymes have been published thus far.

We detected 230 clusters representing S1 peptidases of subfamily S1A, which is typified by chymotrypsin and trypsin. We assigned 216 clusters to 62 peptidase species, whereas 14 clusters represented nonpeptidase homologs (Table 7). Interestingly, only 21 of the 62 species have already been provisionally identified and associated with specific functions, whereas the remaining 41 putative peptidases have not been characterized. Among the identified peptidases, we detected 39 clusters in 7 peptidase species encoding for trypsin-like peptidases (S01.110, S01.116, S01.117, S01.130, S01.A83, S01.A87, and S01.A91). These were mainly expressed in the larval gut (Additional file 6) and probably function as digestive enzymes as reported for other insect species [111]. We provisionally identified two chymotrypsin-like peptidase species, namely, chymotrypsin m-type 2 (S01.168) and Jonah 65Aiv (S01.B05), both of which were strongly expressed in the gut (Additional file 6) as discussed in more detail below. We also identified 8 peptidase species (S01.203, S01.413, S01.421, S01.493, S01.502, S01.507, S01.960, and S01.B27) with different tissue-specific expression profiles (Additional file 6) representing immunity-related peptidases. Melanization peptidase (S01.203), prophenoloxidase-activating peptidase (S01.413), and CLIP-domain prophenoloxidase-activating factor (S01.960) are involved in the regulation of invertebrate innate defenses [170]. Proteolytic lectin (S01.493) was first identified in* Glossina* spp. where it regulates interactions with trypanosome parasites [173]. TmSPE peptidase (S01.507) [174], Persephone (S01.421) [175], Grass (S01.502), and Spirit (S01.B27) [176] facilitate the activation of Toll pathway signaling, which triggers the synthesis of antimicrobial peptides in response to fungi and Gram-negative bacteria [177]. We also identified ovochymase (S01.024), which was discovered in* Xenopus laevis* eggs and may play a role in fertilization or early development [178], the Easter peptidase (S01.201) required for dorsoventral patterning in* D. melanogaster* embryos [179], the Tequila peptidase (S01.461) that mediates long term memory formation in* D. melanogaster* [180], the proapoptotic DmHtrA2-type mitochondrial peptidase (S01.476) [181], and testis-specific protein 50 (S01.993), which is necessary for spermatogenesis in mammals and is upregulated in breast cancer [182].

All the previously identified* L. sericata* serine peptidase genes were also identified in the transcriptome dataset (Table 8). Interestingly, only four of these previously described genes could be assigned to peptidase species with a known function, whereas most were identified based on homology to putative proteins in* D. melanogaster* (Table 8). We also found that although many of the enzymes were detected in MEPs, the corresponding mRNA was predominantly expressed in the larval gut (Additional file 6). The same phenomenon was confirmed for the Jonah-like peptidase, where high expression level of* Jonah* mRNA was observed in the gut but the native enzyme was only detected in MEPs [17]. These results indicate that peptides in MEPs are not exclusively produced by the salivary glands but rather a combination of the salivary glands, gut, and crop. Although further studies are required to confirm this hypothesis, we suggest that regurgitation and/or vomiting in dipteran species [183] contributes to the production of beneficial MEP molecules in* L. sericata* larvae.

Cluster LST_LS005873 was tentatively identified as chymotrypsin m-type 2 (S01.168) and this is identical to the previously described* L. sericata* chymotrypsin I [GenBank: CAS92770]. A recombinant form of this enzyme was shown to degrade wound eschar* ex vivo* [15] and to degrade microbial surface components recognizing adhesive matrix molecules from the slough [184]. As shown in Additional file 6, we identified seven further clusters representing the same peptidase species (S01.168). Another recombinant serine peptidase known as sericase [GenBank: AAA17384] was shown to enhance fibrinolysis [44]. Sericase was found to be identical to* L. sericata* trypsin-like serine peptidase, which was proposed to facilitate wound debridement [185]. We identified three clusters (LST_LS007476, LST_LS007613, and LST_LS010750) homologous to sericase, which represent one peptidase species provisionally identified as* D. melanogaster* putative protein CG7542 (S01.B07). Moreover, the most prominent cluster (LST_LS007476) was also found to be homologous to a previously identified serine peptidase [GenBank: FG360529] which is induced at the transcriptional level following an immune challenge [18]. Our data indicate that sericase is probably involved in several MEP functions including fibrinolysis, debridement, and other immune responses.

Debrilase [GenBank: AJN88395] is a serine peptidase known to play a role in* L. sericata* MDT. Debrilase is homologous to cluster LST_LS015273, which along with another eight clusters represents one peptidase species provisionally identified as* D. melanogaster* putative protein CG17571 (S01.A85). All the clusters share the same tissue-specific expression profile with strong upregulation in the gut (Additional file 6). Recently,* L. sericata* MEPs were shown to reduce the clotting time of human plasma, and this phenomenon was attributed to a serine peptidase activity [45]. Recombinant Jonah-like chymotrypsin was confirmed to reduce the clotting time of human plasma and to degrade certain extracellular matrix proteins [17]. We found 10 clusters representing one peptidase species, tentatively identified as Jonah 65Aiv (S01.B05). These clusters were predominantly expressed in the gut (Additional file 6). Interestingly, cluster LST_LS015269 was found to be homologous to a* L. sericata* serine peptidase [GenBank: FG360505] that is upregulated in immune challenged larvae thus indicating a role in immunity [18].

#### 3.9.2. Family S8

Family S8 comprises two subfamilies of enzymes. Subfamily S8a is typified by the endopeptidase subtilisin, originally identified in* Bacillus subtilis* [69], as well as tripeptidyl-peptidase II, an exopeptidase involved in general intracellular protein turnover [69]. Subfamily S8b is typified by kexin (whose mammalian homolog is known as furin), which processes numerous proteins ranging from growth factors and chemokines to extracellular matrix proteins [186], and is therefore associated with diseases such as Alzheimer's disease, atherosclerosis, and cancer [187]. We identified three clusters of* L. sericata* subtilisin-like enzymes, two clusters similar to tripeptidyl-peptidase II and two clusters as furin-like enzymes (Table 7).

#### 3.9.3. Family S9

The family S9 prolyl oligopeptidases are intracellular enzymes that strictly cleave substrates containing proline residues, and they are thought to process neuropeptides in humans [188]. Interestingly, a prolyl oligopeptidase was recently identified in the human parasite* Schistosoma mansoni*. Although the enzyme is not secreted by the parasite, it cleaves the human vasoregulatory peptides bradykinin and angiotensin I* in vitro*, thus potentially modulating or dysregulating homeostasis in its host [189]. We identified 9 clusters and 4 peptidase species representing* L. sericata* prolyl oligopeptidases. The clusters showed different tissue-specific expression profiles but three of them (LST_LS016452, LST_LS001966, and LST_LS016700) were predominantly expressed in the gut and/or the salivary glands (Additional file 6). This specific distribution in* L. sericata* tissues associated with the production of MEPs indicates a potential role in wound homeostasis but more detailed experiments are required to confirm this hypothesis.

#### 3.9.4. Family S10

Family S10 comprises lysosomal carboxypeptidases with predominantly regulatory functions, although hemipteran S10 peptidases were recently shown to be involved in the digestion of food [152]. Among four family S10 clusters identified in* L. sericata*, two (LST LS003337 and LST_LS015778) were strongly upregulated in the gut, whereas the others (LST_LS004162 and LST_LS016840) were expressed at similar levels in the* L. sericata* tissues we investigated. The clusters induced in the gut were assigned to the peptidase species without a known function, whereas the other two were annotated as vitellogenic carboxypeptidase-like proteins, suggesting a role in vitellogenesis.

#### 3.9.5. Families S14 and S41

Family S14 comprises cytosolic ATP-dependent Clp endopeptidases and their homologs. Clp peptidases together with their ATP-binding subunits create an oligomeric complex of 20–26 subunits [69] that mediate protein quality control and regulatory degradation [190]. Family S41 comprises endopeptidases that are involved in the degradation of incorrectly synthesized proteins. They possess the catalytic tetrad Ser–His–Ser–Glu, which is unusual for serine peptidase, and neither the position of the active site residues nor the residues themselves are conserved [69]. We found that the* L. sericata* families S14 (one cluster in one peptidase species) and S41 (three clusters in two peptidase species) endopeptidases were similarly expressed in all the tissues we analyzed and are likely to be involved in the regulation of protein synthesis.

#### 3.9.6. Family S16

The family S16 enzyme Lon is a bacterial ATP-dependent endopeptidase containing a peptidase domain and an ATP-binding domain in a single subunit. Similar enzymes are found in many other organisms [109] where they facilitate the degradation of unfolded proteins [191]. We identified three clusters assigned to two peptidase species, which were present in all* L. sericata* tissues.

#### 3.9.7. Family S26

Family S26 consists of ubiquitous, membrane-bound enzymes with a catalytic dyad, which are involved in the cleavage of signal peptides thus facilitating the secretion of proteins [69]. We identified two* L. sericata* family S26 clusters and two peptidase species representing signal peptidases and another cluster that remained unassigned (Table 7). Cluster LST_LS009731 was strongly expressed in the salivary glands, which are known to secret large amounts of protein, thus indicating a role in protein secretion.

#### 3.9.8. Family S28

Family S28 comprises the lysosomal Pro-Xaa carboxypeptidases, which are lysosome-specific exopeptidase found solely in eukaryotes, featuring an unusual selectivity for the cleavage of ProXaa bonds. In humans, such enzymes inactivate angiotensin II and activate plasma kallikrein [192]. We identified three* L. sericata* family S28 clusters assigned to two peptidase species that were upregulated in the crop and larval body samples (Additional file 6). The precise role of these enzymes remains unclear although they may contribute to the procoagulation activity of MEPs as recently described [45].

#### 3.9.9. Family S49

Only one cluster in one peptidase species was assigned to the family S49. Family S49 is the signal peptidases required for intracellular protein processing and the regulation of protein export [193].

#### 3.9.10. Family S51

Family S51 is typified by aspartyl dipeptidase, an exopeptidase originally identified in* Salmonella typhimurium* [194] that hydrolyzes *α*-aspartyl bonds. The crystal structure of the* S. typhimurium* aspartyl dipeptidase has been solved, revealing an unusual catalytic triad with Ser and His as predicted but Glu instead of Asp [195]. The biological role for this enzyme is not clear, but it seems to be involved in the production of nutritional amino acids [69]. Two clusters belonging to one peptidase species were identified in* L. sericata* transcriptome.

#### 3.9.11. Family S54

Family S54 is typified by Rhomboid-1, an intramembrane enzyme identified in* D. melanogaster* [196] that plays an important role in embryonic development by cleaving the Spitz protein and thus activating the epidermal growth factor receptor [197]. We identified three clusters in three peptidase species, which were expressed in all* L. sericata* tissues.

### 3.10. qRT-PCR Verification of Gene Expression

**Figure 6:**
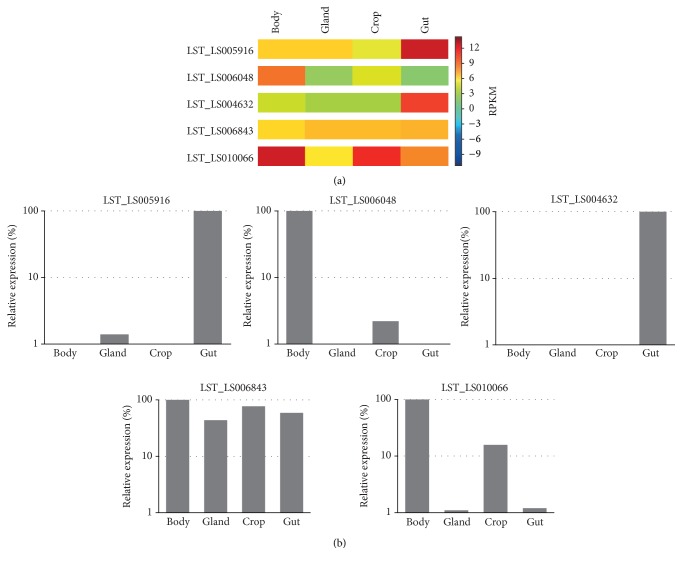
Verification of digital gene expression analysis by quantitative RT-PCR. (a) The individual clusters encoding for aspartic (LST_LS005916), cysteine (LST_LS006048), metallo (LST_LS004632), threonine (LST_LS006843), and serine (LST_LS010066) peptidases are depicted on the left. Shown are log_2_-transformed RPKM values (blue resembles lower-expressed genes, while red represents highly expressed genes). (b) The mRNA expression profiles of five enzymatic genes in various larval tissues were determined by qRT-PCR and normalized with the* 60S acidic ribosomal protein P0* (RPLPO) and* 40S ribosomal protein S3* (RPS3) genes. The expressions of individual genes were related to the maximum mRNA level of each gene set as 100%.

To experimentally verify the results from digital gene expression analysis we performed qRT-PCR analysis of one peptidase gene from each peptidase clan (aspartic, cysteine, metallo, threonine, and serine). These genes (Table 9) code for peptidases with various physiological function and show different tissue expression profile. All the tested genes show the similar expression profiles as acquired by digital gene expression analysis (Figure 6).

## 4. Conclusion

The purpose of this study was to provide an overview of the distribution of proteolytic enzymes in* L. sericata*, focusing on the tissue-specific expression profiles and potential functions as the basis for further more detailed studies of individual peptidases. We identified 577 clusters representing five classes of proteolytic enzymes (aspartic, cysteine, threonine, serine, and metallopeptidases) which were further assigned into 26 clans, 48 families, and 185 peptidase species with diverse tissue-specific patterns of distribution. We identified all previously described therapeutic peptidases and found that most of them were most highly expressed in the larval gut, thus indicating that the larval gut contributes to the production of beneficial enzymes found in the MEPs. Although the majority of the enzymes we identified were serine peptidases, most of them were novel putative peptidases whose function is unclear, but whose specific tissue-specific expression profiles indicate an important role in MEP activity. Several peptides with the most intriguing expression profiles have been prepared as synthetic genes allowing the functional analysis of the corresponding recombinant peptides.

## Supplementary Material

The supplementary table 1 shows number of L. sericata transcripts assembled with Trinity and Oases. K-mer sizes vary between 21 and 75 for the Oases assembly. The supplementary figures 1 to 5 show Heat maps with relative expression levels of all L. sericata peptidases. The individual clusters encoding for aspartic (Fig1), cysteine (Fig2), metallo (Fig3), threonine (Fig4) and serine (Fig5) peptidases are depicted on the left, while corresponding clans, families and MEROPS IDs are depicted on the right. Shown are log2-transformed RPKM values (blue resembles lower-expressed genes, while red represents highly expressed genes). The supplementary figure 6 shows the melting curve analysis of all genes which were tested via qRT-PCR.

## Figures and Tables

**Table 1:** Illumina sequencing data representing different *L. sericata* tissues.

	Reads	Bases
Body	29,536,054	4.38 Gb
Crop	26,402,527	3.96 Gb
Salivary glands	34,206,636	5.09 Gb
Gut	33,711,437	5.09 Gb

**Table 2:** General overview of *L. sericata* peptidases.

Class	Clans	Families	Species	Unassigned peptidases	Nonpeptidase homologs
Aspartic peptidase	2	3	7	1	—
Cysteine peptidase	6	11	23	8	1
Metallopeptidase	9	20	53	12	9
Threonine peptidase	1	2	16	—	—
Serine peptidase	8	12	86	2	1
Total	**26**	**48**	**185**	**23**	**11**

**Table 3:** List of aspartic peptidases in the *L. sericata* transcriptome database.

Clan	Family	Peptidase type	Peptidase species	MEROPS ID	Number of clusters
AA	A1	Pepsin A	CAD2 peptidase (*M. domestica*)	A01.092	5
			CAD3 peptidase (*M. domestica*)	A01.093	2
			CG10104 g.p. (*D. melanogaster*)	A01.A64	2
			CG17134 protein (*D. melanogaster*)	A01.A78	1

AD	A22	Presenilin 1	impas 1 peptidase	A22.003	2
			impas 2 peptidase	A22.005	1
			Psn peptidase (*Drosophila*-type)	A22.014	1

AA	A28	DNA-damage inducible protein 1	Family A28 unassigned peptidases	A28.UPW	1

**Table 4:** List of cysteine peptidases in the *L. sericata* transcriptome database.

Clan	Family	Peptidase type	Peptidase species	MEROPS ID	Number of clusters
CA	C1	Papain	Insect 26/29 kDa peptidase	C01.067	2
			Vitellogenic cathepsin B	C01.068	1
			Bleomycin hydrolase (animal)	C01.084	2
			Papain homologue (Rattus-type)	C01.107	3
			Cathepsin B-like cysteine peptidase (Raphanus sativus)	C01.144	1
			CG12163 protein (*D. melanogaster*)	C01.A27	2
			CG4847 protein (*D. melanogaster*)	C01.A28	1
			Nonpeptidase homologues	C01.UNA	3
			Subfamily C1A unassigned peptidases	C01.UPA	1

CA	C2	Calpain 2	Calpain-15	C02.010	1
			Calpain A	C02.14	1

CA	C12	Ubiquitinyl hydrolase-L1	Ubiquitinyl hydrolase-L5	C12.005	1
			CG8445 protein (*D. melanogaster*)	C12.A09	1
			Family C12 unassigned peptidases	C12.UPW	1

CA	C54	Autophagin-1	CG4428 protein (*D. melanogaster*)	C54.A04	1
			Family C54 unassigned peptidases	C54.UPW	2

CA	C65	Otubain-1	Otubain-1	C65.001	1

CD	C13	Legumain	Glycosylphosphatidylinositol:protein transamidase	C13.005	2
			Family C13 unassigned peptidases	C13.UPW	2

CD	C14	Caspase-1	Caspase (insect 1)	C14.015	2
			Caspase (insect 2)	C14.016	1
			Caspase DRONC- (*D. melanogaster*-) type peptidase	C14.019	2
			Caspase DECAY	C14.022	1
			Caspase STRICA (*Drosophila* sp.)	C14.023	1
			Caspase Dredd	C14.040	1

CE	C48	Ulp1 peptidase	Ulp1 peptidase (*Drosophila*-type)	C48.024	1
			Family C48 unassigned peptidases	C48.UPW	1

CF	C15	Pyroglutamyl-peptidase	Family C15 unassigned peptidases	C15.UPW	1

PC	C26	Gamma-glutamyl hydrolase	CG32155 protein (*D. melanogaster*)	C26.A22	2
			Family C26 unassigned peptidases	C26.UPW	1

PD	C46	Hedgehog protein	Hedgehog protein	C46.001	1
			Family C46 unassigned peptidases	C46.UPW	1

**Table 5:** List of metallopeptidases in the *L. sericata* transcriptome database.

Clan	Family	Peptidase type	Peptidase species	MEROPS ID	Number of clusters
MA	M1	Aminopeptidase N	ERAP2 aminopeptidase	M01.024	5
			Slamdance (*D. melanogaster*)	M01.A02	2
			CG5845 protein (*D. melanogaster*)	M01.A05	1
			CG11956 protein (*D. melanogaster*)	M01.A09	6
			Dgri protein (*D. melanogaster*)	M01.A10	2
			CG8774 protein (*D. melanogaster*)	M01.A11	3
			CG8773 protein (*D. melanogaster*)	M01.A12	1
			AT4g33090 g.p. (*Arabidopsis thaliana*)	M01.A25	6
			Family M1 nonpeptidase homologues	M01.UNW	3
			Family M1 unassigned peptidases	M01.UPW	4

MA	M2	Angiotensin-converting enzyme peptidase unit 1	Peptidyl-dipeptidase Ance	M02.003	16
			Family M2 nonpeptidase homologues	M02.UNW	6
			Family M2 unassigned peptidases	M02.UPW	4

MA	M3	Thimet oligopeptidase	Subfamily M3A nonpeptidase homologues	M03.UNA	1
			Subfamily M3A unassigned peptidases	M03.UPA	1

MA	M8	Leishmanolysin	Family M8 unassigned peptidases	M08.UPW	2

MA	M10	Matrix metallopeptidase-1	Dm1 matrix metallopeptidase	M10.031	2
			Dm2-matrix metallopeptidase	M10.036	2

MA	M12	Astacin	Mammalian-type tolloid-like 2 protein	M12.018	1
			ADM-4 peptidase (*Caenorhabditis elegans*)	M12.329	1
			ADAMTS-A (*D. melanogaster*)	M12.A03	1
			CG4096 protein (*D. melanogaster*)	M12.A04	2
			CG4096 protein (*D. melanogaster*)	M12.A05	1
			CG6696 protein (*D. melanogaster*)	M12.A08	1
			CG15254 protein (*D. melanogaster*)	M12.A09	1
			Subfamily M12A unassigned peptidases	M12.UPA	10
			Subfamily M12B unassigned peptidases	M12.UPB	8

MA	M13	Neprilysin	Zmp1 peptidase (*Mycobacterium*-type)	M13.009	2
			Nep2 peptidase (insect)	M13.012	2
			Neprilysin-4 (*D. melanogaster*)	M13.014	2
			CG3775 protein (*D. melanogaster*)	M13.A01	2
			CG14526 protein (*D. melanogaster*)	M13.A04	2
			CG14528 protein (*D. melanogaster*)	M13.A06	1
			CG9507 protein (*D. melanogaster*)	M13.A11	2
			CG5894 protein (*D. melanogaster*)	M13.A15	2
			Family M13 nonpeptidase homologues	M13.UNW	6
			Family M13 unassigned peptidases	M13.UPW	13

MA	M41	FtsH peptidase	Paraplegin	M41.006	1
			YME1L1 g.p. (*Homo sapiens*) and similar	M41.026	1
			Family M41 nonpeptidase homologues	M41.UNW	1

MA	M48	Ste24 peptidase	CG9001 protein (*D. melanogaster*)	M48.A06	2

MA	M49	Dipeptidyl-peptidase III	CG7415 g.p. (*D. melanogaster*)	M49.A01	2

MC	M14	Carboxypeptidase A1	CG8560 protein (*D. melanogaster*)	M14.A04	2
			CG2915 protein (*D. melanogaster*)	M14.A05	6
			CG17633 protein (*D. melanogaster*)	M14.A08	1
			CG14820 protein (*D. melanogaster*)	M14.A12	2
			CG8562 protein (*D. melanogaster*)	M14.A15	1
			CG18417 protein (*D. melanogaster*)	M14.A16	1
			CG3097 protein (*D. melanogaster*)	M14.A19	1
			Silver protein domain 2 (*D. melanogaster*)	M14.A20	2
			CG11428 (*D. melanogaster*)	M14.A21	2
			Subfamily M14A nonpeptidase homologues	M14.UNA	1
			Subfamily M14A unassigned peptidases	M14.UPA	11
			Subfamily M14B unassigned peptidases	M14.UPB	3

ME	M16	Pitrilysin	Mitochondrial processing peptidase beta-subunit	M16.003	1
			Eupitrilysin	M16.009	1
			Mername-AA224 nonpeptidase homologue	M16.975	2
			CG2025 protein (*D. melanogaster*)	M16.A06	3
			C02G6.1 g.p. (*C. elegans*)	M16.A08	1
			LOC133083 g.p. (*H. sapiens*)	M16.P01	2

MF	M17	Leucine aminopeptidase 3	Leucyl aminopeptidase-1 (*Caenorhabditis*-type)	M17.006	2
			Leucine aminopeptidase (*Fasciola*-type)	M17.011	2

MG	M24	Methionyl aminopeptidase 1	Xaa-Pro dipeptidase (eukaryote)	M24.007	2
			Methionyl aminopeptidase 1 (eukaryote)	M24.017	1
			Subfamily M24B nonpeptidase homologues	M24.UNB	1
			Family M24 nonpeptidase homologues	M24.UNW	1
			Subfamily M24A unassigned peptidases	M24.UPA	2
			Subfamily M24B unassigned peptidases	M24.UPB	2

MH	M20	Glutamate carboxypeptidase	Peptidase T	M20.003	1

	M28	Aminopeptidase S	Aminopeptidase ES-62 (*Acanthocheilonema viteae*)	M28.A15	1
			Family M28 unassigned peptidases	M28.UPW	6

MJ	M19	Membrane dipeptidase	Family M19 nonpeptidase homologues	M19.UNW	1

MM	M50	Site 2 peptidase	dS2P peptidase (*D. melanogaster*)	M50.007	1

MP	M67	RPN11 peptidase	At1g71230 (*A. thaliana*)	M67.A02	1

UNA	M79	RCE1 peptidase	RCE1 peptidase (*H. sapiens*-type)	M79.002	1

**Table 6:** List of threonine peptidases in the *L. sericata* transcriptome database.

Clan	Family	Peptidase type	Peptidase species	MEROPS ID	Number of clusters
PB	T1	Archaean proteasome, beta component	Constitutive proteasome catalytic subunit 1	T01.010	2
			Proteasome subunit alpha 6	T01.971	2
			Proteasome subunit alpha 2	T01.972	1
			Proteasome subunit alpha 4	T01.973	2
			Proteasome subunit alpha 7	T01.974	2
			Proteasome subunit alpha 5	T01.975	2
			Proteasome subunit alpha 1	T01.976	1
			Proteasome subunit alpha 3	T01.977	2
			Proteasome subunit beta 2	T01.984	1
			Proteasome subunit beta 1	T01.986	1
			Proteasome subunit beta 4	T01.987	2
			Proteasome subunit beta2 (*D. melanogaster*)	T01.A02	1
			Beta 5 subunit (*D. melanogaster*)	T01.A06	1
			Mername-AA231 pseudogene (*H. sapiens*)	T01.P02	1

PB	T2	Glycosylasparaginase precursor	Isoaspartyl dipeptidase (threonine type)	T02.002	3
			Taspase-1	T02.004	1

**Table 7:** List of serine peptidases in the *L. sericata* transcriptome database.

Clan	Family	Peptidase type	Peptidase species	MEROPS ID	Number of clusters
PA	S1	Chymotrypsin A	Ovochymase domain 2 (*X. laevis*)	S01.024	3
			Trypsin alpha (insect) (*D. melanogaster*)	S01.110	23
			Trypsin zeta (insect) (*D. melanogaster*)	S01.116	7
			Trypsin eta (insect) (*D. melanogaster*)	S01.117	3
			Trypsin (mosquito type) (*Anopheles gambiae*)	S01.130	2
			Chymotrypsin m-type 2 (insect) (*Lucilia cuprina*)	S01.168	7
			Easter peptidase (*D. melanogaster*)	S01.201	6
			Melanization peptidase-2 (*D. melanogaster*)	S01.203	2
			CG4386 peptidase (*Sarcophaga peregrina*)	S01.316	2
			Prophenoloxidase-activating peptidase (*Pacifastacus leniusculus*)	S01.413	2
			Persephone peptidase (*D. melanogaster*)	S01.421	1
			Tequila peptidase (*D. melanogaster*)	S01.461	2
			DmHtrA2-type peptidase (*M. domestica*)	S01.476	2
			Proteolytic lectin (*Glossina*-type) (*Glossina fuscipes fuscipes*)	S01.493	3
			Grass peptidase (Insecta) (*D. melanogaster*)	S01.502	3
			TmSPE peptidase (*Tenebrio*-type) (*Tenebrio molitor*)	S01.507	2
			CLIP-domain prophenoloxidase activating factor (*Cotesia rubecula*)	S01.960	8
			Testis-specific protein 50 (*H. sapiens*)	S01.993	1
			CG34409 protein (*D. melanogaster*)	S01.A23	1
			CG11670 protein (*D. melanogaster*)	S01.A31	1
			CG14780 protein (*D. melanogaster*)	S01.A35	1
			CG8952 protein (*D. melanogaster*)	S01.A36	2
			CG8170 protein (*D. melanogaster*)	S01.A37	1
			CG2105 (*D. melanogaster*)	S01.A51	2
			Sp212 protein (*D. melanogaster*)	S01.A52	1
			CG9733 protein (*D. melanogaster*)	S01.A61	2
			CG7829 protein (*D. melanogaster*)	S01.A62	1
			CG5909 protein (*D. melanogaster*)	S01.A64	1
			CG6048 protein (*D. melanogaster*)	S01.A77	3
			CG6041 protein (*D. melanogaster*)	S01.A78	4
			Trypsin beta (*D. melanogaster*)	S01.A83	1
			CG17571 protein (*D. melanogaster*)	S01.A85	9
			Trypsin lambda (*D. melanogaster*)	S01.A87	2
			CG9676 protein (*D. melanogaster*)	S01.A89	7
			Try29F protein (*D. melanogaster*)	S01.A91	1
			CG17477 protein (*D. melanogaster*)	S01.A92	1
			CG5246 protein (*D. melanogaster*)	S01.A94	7
			CG17475 protein (*D. melanogaster*)	S01.A96	1
			CG5233 protein (*D. melanogaster*)	S01.A97	2
			Jonah 65Aiv (*D. melanogaster*)	S01.B05	10
			CG7542 protein (*D. melanogaster*)	S01.B07	3
			CG10477 protein (*D. melanogaster*)	S01.B12	19
			CG6457 protein (*D. melanogaster*)	S01.B13	2
			CG6483 protein (*D. melanogaster*)	S01.B14	1
			CG10472 protein (*D. melanogaster*)	S01.B16	2
			CG11529 protein (*D. melanogaster*)	S01.B22	1
			IP21814 protein (*D. melanogaster*)	S01.B25	2
			Spirit protein (*D. melanogaster*)	S01.B27	4
			CG3700 protein (*D. melanogaster*)	S01.B30	2
			CG1299 protein (*D. melanogaster*)	S01.B33	12
			CG34350 protein (*D. melanogaster*)	S01.B35	1
			CG4914 protein (*D. melanogaster*)	S01.B41	1
			CG11836 protein (*D. melanogaster*)	S01.B43	1
			CG16821 protein (*D. melanogaster*)	S01.B44	1
			CG32808 protein (*D. melanogaster*)	S01.B47	1
			CG16749 protein (*D. melanogaster*)	S01.B48	5
			CG8299 protein (*D. melanogaster*)	S01.B50	2
			IP10861 protein (*D. melanogaster*)	S01.B61	1
			CG16996 protein (*D. melanogaster*)	S01.B64	2
			CG7142 protein (*D. melanogaster*)	S01.B65	1
			CG11911 protein (*D. melanogaster*)	S01.B68	8
			CG10663-PC, isoform C (*D. melanogaster*)	S01.B77	4
			Subfamily S1A non-peptidase homologues	S01.UNA	14

SB	S8	Subtilisin Carlsberg	Furin-1 (arthropod-type)	S08.048	1
			Furin-2 (insect)	S08.049	1
			Site-1 peptidase	S08.063	1
			KPC2-type peptidase	S08.109	2
			Subfamily S8A unassigned peptidases	S08.UPA	2

SC	S9	Prolyl oligopeptidase	Omega peptidase (*D. melanogaster*)	S09.069	2
			CG11034 protein (*D. melanogaster*)	S09.A64	2
			CG11319 (*D. melanogaster*)	S09.A65	4
			Xaa-Pro dipeptidylpeptidase	S09.A73	1

SC	S10	Carboxypeptidase Y	Vitellogenic carboxypeptidase-like protein	S10.003	2
			CG32483 protein (*D. melanogaster*)	S10.A52	2

SC	S28	Lysosomal Pro-Xaa carboxypeptidase	Lysosomal Pro-Xaa carboxypeptidase	S28.001	1
			CG9953 protein (*D. melanogaster*)	S28.A10	2

SF	S26	Signal peptidase I	Signal peptidase (invertebrate)	S26.023	1
			CG11110 protein (*D. melanogaster*)	S26.A09	1
			Subfamily S26A unassigned peptidases	S26.UPA	1

SJ	S16	Lon-A peptidase	Lon-A peptidase	S16.001	2
			PIM1 peptidase	S16.002	1

SK	S14	Peptidase Clp	Peptidase Clp (type 3)	S14.003	1

SK	S41	C-terminal processing peptidase-1	C-terminal processing peptidase-1	S41.001	1
			C-terminal processing peptidase-2	S41.004	2

SK	S49	Signal peptide peptidase A	BSn5_05605 g.p. (*Bacillus subtilis*)	S48.A08	1

ST	S54	Rhomboid-1	Rhomboid-1 (Diptera)	S54.001	1
			Rhomboid-4 (insect)	S54.012	2
			Cg8972 protein	S54.A12	1

PC	S51	Dipeptidase E	Alpha-aspartyl dipeptidase (eukaryote)	S51.002	2

**Table 8:** Comparison of clusters with previously identified *L. sericata* S1 peptidases.

GenBank ID	Name	Proposed function	Reference	Cluster	Peptidase species	MEROPS ID
CAS92770	Chymotrypsin I	Wound debridement	[15]	LST_LS005873	Chymotrypsin m-type 2 (insect) (*L. cuprina*)	S01.168

KT158461	Jonah-like chymotrypsin	Plasma clotting/debridement	[17]	LST_LS007060	Jonah 65Aiv (*D. melanogaster*)	S01.B05

AAA17384	Sericase/trypsin	Fibrinolysis/debridement	[44]	LST_LS007476	CG7542 protein (*D. melanogaster*)	S01.B07
			[185]		CG7542 protein (*D. melanogaster*)	S01.B07

AEX33291	Putative salivary trypsin	Not described	[198]	LST_LS016882	CLIP-domain prophenoloxidase activating factor (*C. rubecula*)	S01.960
				LST_LS005035	CLIP-domain prophenoloxidase activating factor (*C. rubecula*)	S01.960
				LST_LS005470	CLIP-domain prophenoloxidase activating factor (*C. rubecula*)	S01.960
				LST_LS016440	CLIP-domain prophenoloxidase activating factor (*C. rubecula*)	S01.960
				LST_LS005552	CLIP-domain prophenoloxidase activating factor (*C. rubecula*)	S01.960
				LST_LS005028	CLIP-domain prophenoloxidase activating factor (*C. rubecula*)	S01.960

AEX33294	Putative salivary trypsin	Not described	[198]	LST_LS003737	IP10861 protein (*D. melanogaster*)	S01.B61

AEX33290	Putative chymotrypsin	Not described	[198]	LST_LS016062	CG10477 protein (*D. melanogaster*)	S01.B12
				LST_LS007621	CG10477 protein (*D. melanogaster*)	S01.B12

AJN88395	Debrilase	Debridement	Patent # US 8623810	LST_LS015273	CG17571 protein (*D. melanogaster*)	S01.A85

FG360474		Upregulated upon immune challenge	[18]	LST_LS007407	CG11911 protein (*D. melanogaster*)	S01.B68
				LST_LS015021	CG11911 protein (*D. melanogaster*)	S01.B68

FG360505		Upregulated upon immune challenge	[18]	LST_LS015269	Jonah 65Aiv (*D. melanogaster*)	S01.B05

FG360512		Upregulated upon immune challenge	[18]	LST_LS008125	Subfamily S1A non-peptidase homologues	S01.UNA

FG360521		Upregulated upon immune challenge	[18]	LST_LS008080	CG9676 protein (*D. melanogaster*)	S01.A89

FG360523		Upregulated upon immune challenge	[18]	LST_LS003034	Trypsin alpha (insect) (*D. melanogaster*)	S01.110

FG360527		Upregulated upon immune challenge	[18]	LST_LS007843	CG17571 protein (*D. melanogaster*)	S01.A85

FG360528		Upregulated upon immune challenge	[18]	LST_LS015518	CG16749 protein (*D. melanogaster*)	S01.B48
				LST_LS007441	CG16749 protein (*D. melanogaster*)	S01.B48

FG360529		Upregulated upon immune challenge	[18]	LST_LS007476	CG7542 protein (*D. melanogaster*)	S01.B07

**Table 9:** Genes and qRT-PCR primers evaluated in this study.

Clan	Family	MEROPS ID	Peptidase sp.	Cluster	Primer sequences (5′- 3′)	*L* (bp)^a^	*E* (%)^b^	*R* ^2^ ^c^
AA	A1	A01.092	CAD2 peptidase (*M. domestica*)	LST_LS005916	5′-GCTGCCAAGCTATTGCTGAT-3′	75	102	0.997
					5′-CGGCTTGAATGTTTTCGTATT-3′			

CA	C1	C01.A27	CG12163 protein (*D. melanogaster*)	LST_LS006048	5′- TTTCACCGGTAATCGCAAAT -3′	66	105	0.997
					5′- GCAGCCTTTTGACGATGTTT -3′			

MA	M1	M01.024	ERAP2 aminopeptidase	LST_LS004632	5′- CCATTCCGTCCGAT TCTT -3′	68	98	0.999
					5′- ATCGGCATACTGGGCAAA -3′			

PB	T1	T01.976	Proteasome subunit alpha 1	LST_LS006843	5′- TCTTCACGCTCTCAAGACGA -3′	61	103	0.993
					5′- GGTCTTGTGTTTCGCCATCT -3′			

PA	S1	S01.993	Testis-specific protein 50 (*H. sapiens*)	LST_LS010066	5′- TATGGGCAGCCCTAACGA -3′	60	104	0.998
					5′- AAAGAACGGAGAGCATCACCT -3′			

^a^Length of amplicon.

^b^Quantitative qRT-PCR efficiency.

^c^Coefficient of determination.

## Additional Points

The dataset supporting the results of this paper is available in the (EBI Short Read Archive [SRA]) repository under the accession number PRJEB7567. The complete study can be accessed directly at http://www.ebi.ac.uk/ena/data/view/PRJEB7567.
