# Supplementary material for: Next Generation Sequencing Identifies Five Major Classes of Potentially Therapeutic Enzymes Secreted by Lucilia sericata Medical Maggots
Source: Biomed Res Int. 2016 Mar 28;2016:8285428. doi: 10.1155/2016/8285428 (PMC4826915; doi:10.1155/2016/8285428)
Supplement: Supplementary file 1 — The supplementary table 1 shows number of L. sericata transcripts assembled with Trinity and Oases. K-mer sizes vary between 21 and 75 for the Oases assembly. The supplementary figures 1 to 5 show Heat maps with relative expression levels of all L. sericata peptidases. The individual clusters encoding for aspartic (Fig1), cysteine (Fig2), metallo (Fig3), threonine (Fig4) and serine (Fig5) peptidases are depicted on the left, while corresponding clans, families and MEROPS IDs are depicted on the right. Shown are log2-transformed RPKM values (blue resembles lower-expressed genes, while red represents highly expressed genes). The supplementary figure 6 shows the melting curve analysis of all genes which were tested via qRT-PCR. [file 8285428.f1.zip › AF1_ assembly.pdf]

Normalized reads assembly using Trinity and 28 individual velvet/oases assemblies

Assembly	Contigs
Trinity	50163
Oases k=21	133837
Oases k=23	116774
Oases k=25	108478
Oases k=27	104756
Oases k=29	99996
Oases k=31	96169
Oases k=33	90999
Oases k=35	84424
Oases k=37	77575
Oases k=39	72446
Oases k=41	67807
Oases k=43	63829
Oases k=45	59249
Oases k=47	55612
Oases k=49	52134
Oases k=51	49783
Oases k=53	47177
Oases k=55	44908
Oases k=57	42841
Oases k=59	40770
Oases k=61	38555
Oases k=63	36350
Oases k=65	34053
Oases k=67	31410
Oases k=69	28641
Oases k=71	25566
Oases k=73	21900
Oases k=75	17943
