# Supplementary figures and images for: Next Generation Sequencing Identifies Five Major Classes of Potentially Therapeutic Enzymes Secreted by Lucilia sericata Medical Maggots
Source: Biomed Res Int. 2016 Mar 28;2016:8285428. doi: 10.1155/2016/8285428 (PMC4826915; doi:10.1155/2016/8285428)

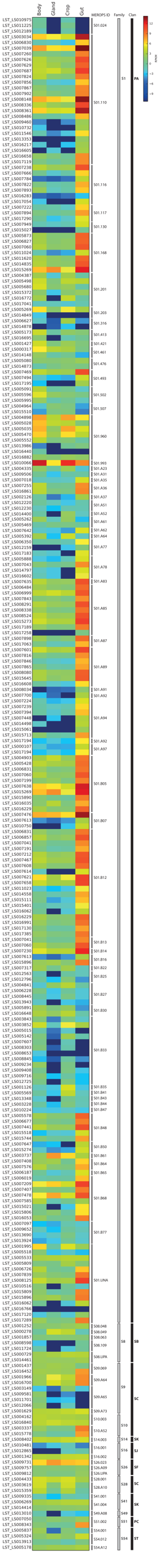

Supplement: Supplementary file 1 — The supplementary table 1 shows number of L. sericata transcripts assembled with Trinity and Oases. K-mer sizes vary between 21 and 75 for the Oases assembly. The supplementary figures 1 to 5 show Heat maps with relative expression levels of all L. sericata peptidases. The individual clusters encoding for aspartic (Fig1), cysteine (Fig2), metallo (Fig3), threonine (Fig4) and serine (Fig5) peptidases are depicted on the left, while corresponding clans, families and MEROPS IDs are depicted on the right. Shown are log2-transformed RPKM values (blue resembles lower-expressed genes, while red represents highly expressed genes). The supplementary figure 6 shows the melting curve analysis of all genes which were tested via qRT-PCR. [file 8285428.f1.zip › AF6_Serine_HM.pdf]

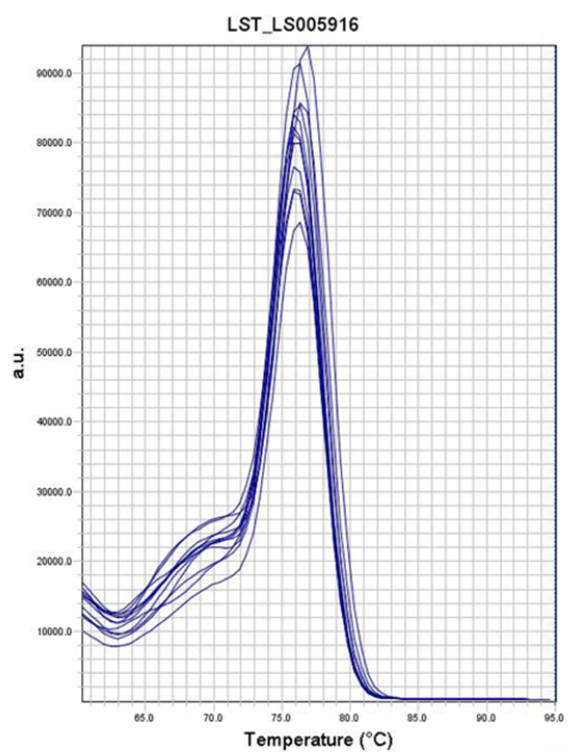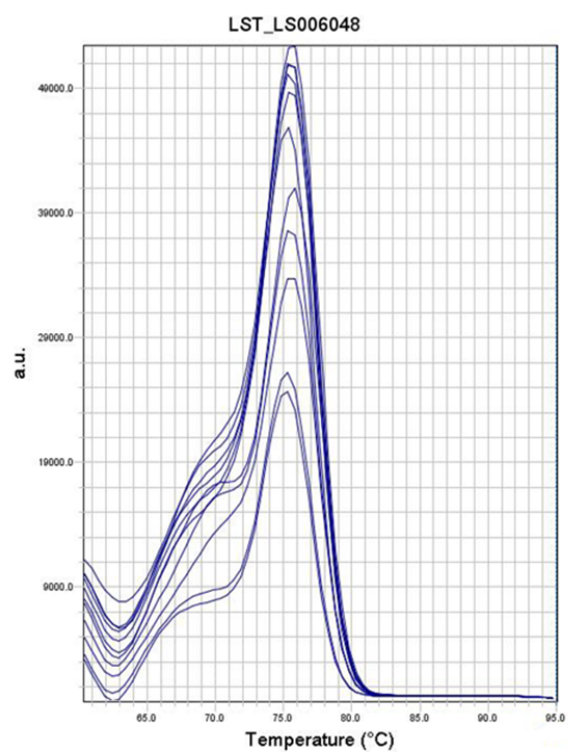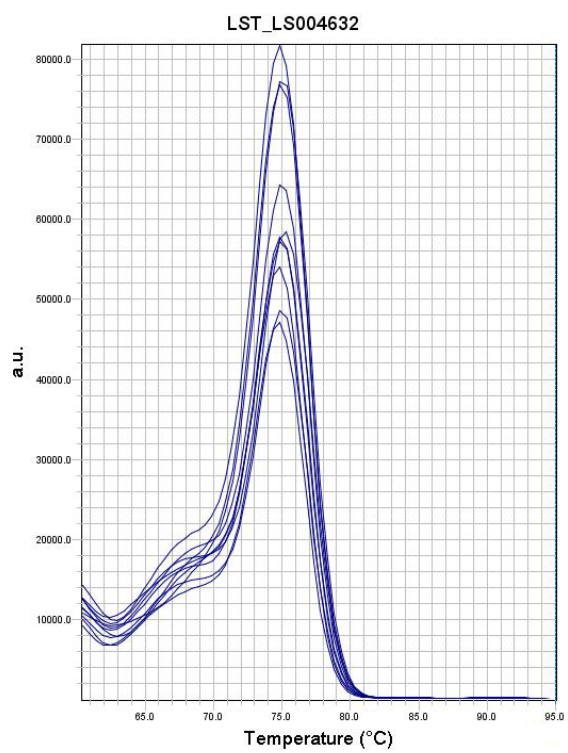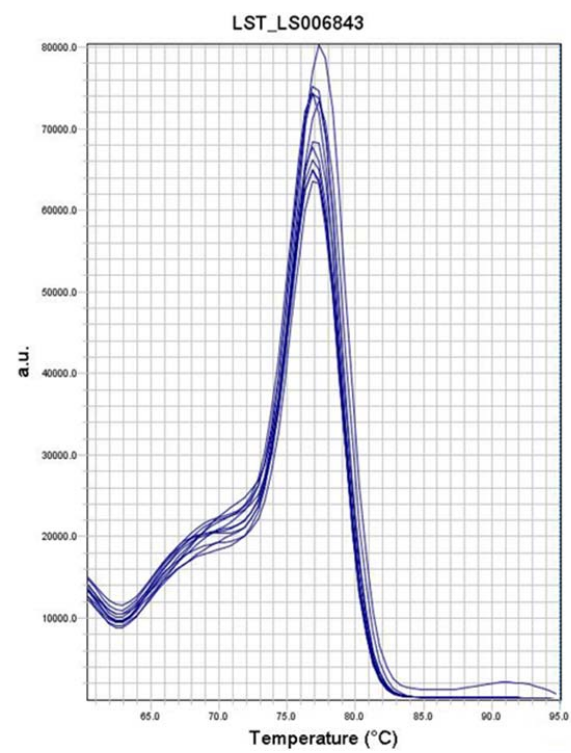

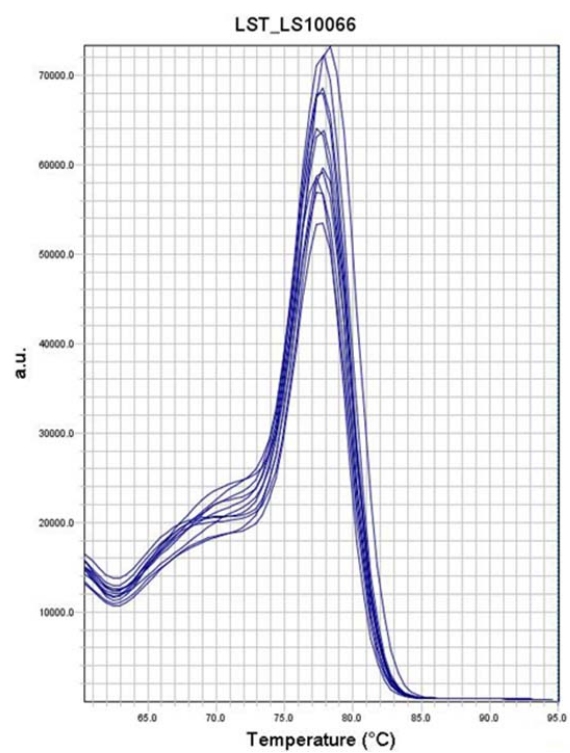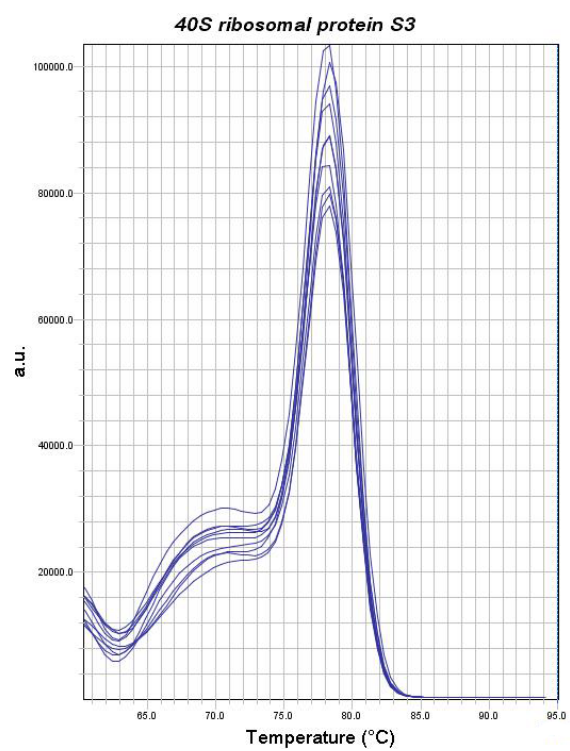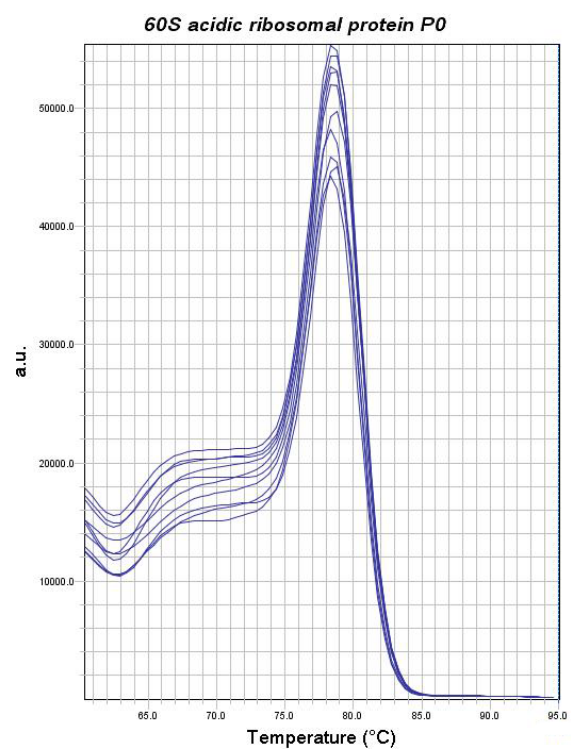

Supplement: Supplementary file 1 — The supplementary table 1 shows number of L. sericata transcripts assembled with Trinity and Oases. K-mer sizes vary between 21 and 75 for the Oases assembly. The supplementary figures 1 to 5 show Heat maps with relative expression levels of all L. sericata peptidases. The individual clusters encoding for aspartic (Fig1), cysteine (Fig2), metallo (Fig3), threonine (Fig4) and serine (Fig5) peptidases are depicted on the left, while corresponding clans, families and MEROPS IDs are depicted on the right. Shown are log2-transformed RPKM values (blue resembles lower-expressed genes, while red represents highly expressed genes). The supplementary figure 6 shows the melting curve analysis of all genes which were tested via qRT-PCR. [file 8285428.f1.zip › AF7_melt curves 5 + 2 ref gen.pdf]

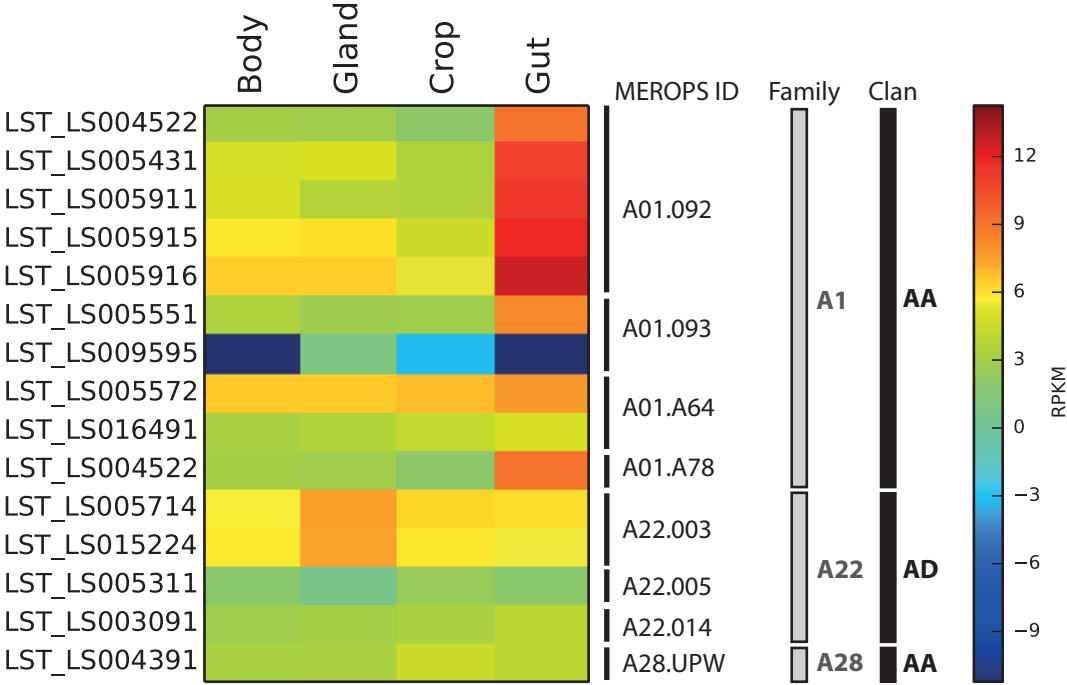

Supplement: Supplementary file 1 — The supplementary table 1 shows number of L. sericata transcripts assembled with Trinity and Oases. K-mer sizes vary between 21 and 75 for the Oases assembly. The supplementary figures 1 to 5 show Heat maps with relative expression levels of all L. sericata peptidases. The individual clusters encoding for aspartic (Fig1), cysteine (Fig2), metallo (Fig3), threonine (Fig4) and serine (Fig5) peptidases are depicted on the left, while corresponding clans, families and MEROPS IDs are depicted on the right. Shown are log2-transformed RPKM values (blue resembles lower-expressed genes, while red represents highly expressed genes). The supplementary figure 6 shows the melting curve analysis of all genes which were tested via qRT-PCR. [file 8285428.f1.zip › AF2_Aspartic_HM.pdf]

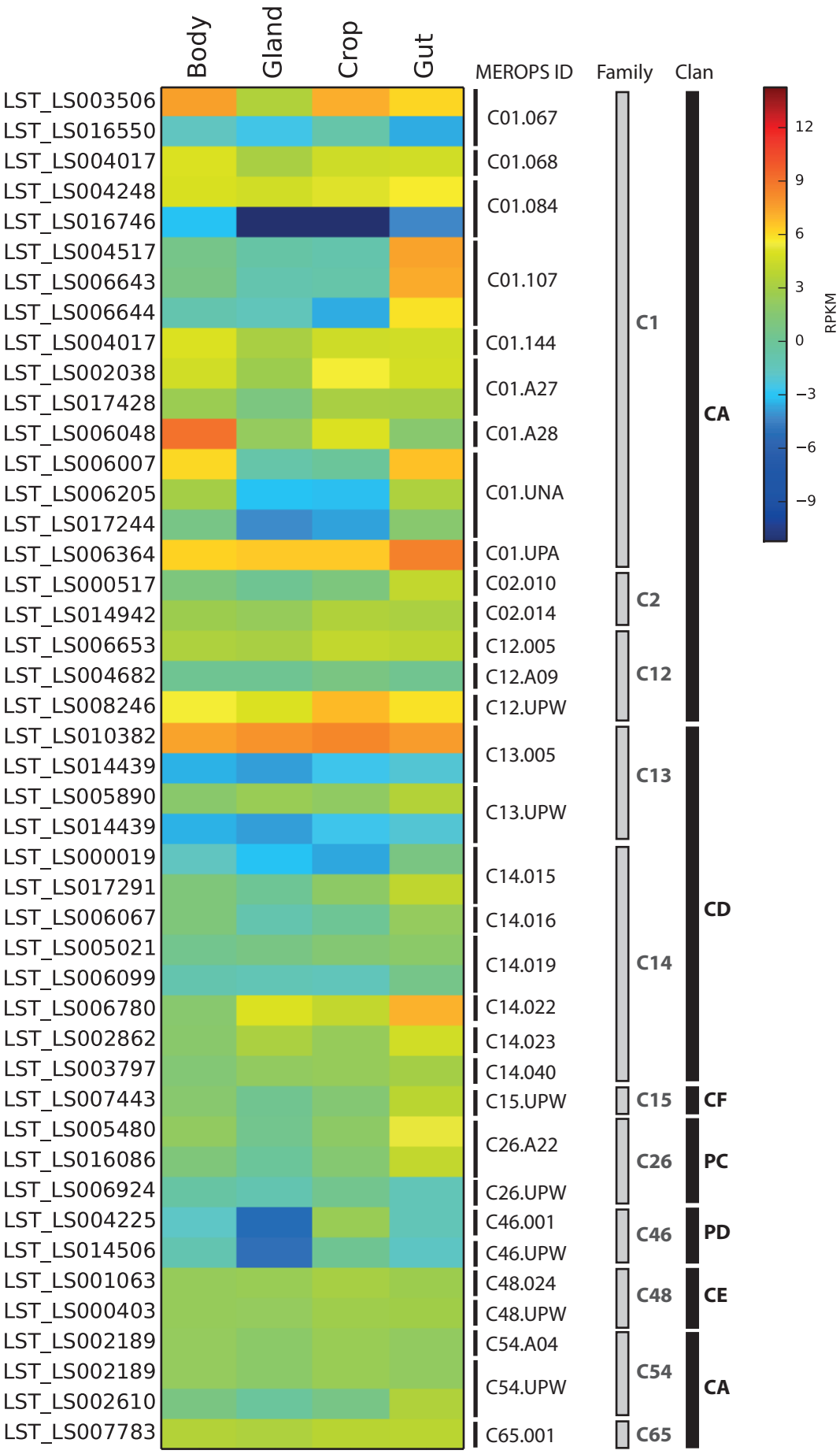

Supplement: Supplementary file 1 — The supplementary table 1 shows number of L. sericata transcripts assembled with Trinity and Oases. K-mer sizes vary between 21 and 75 for the Oases assembly. The supplementary figures 1 to 5 show Heat maps with relative expression levels of all L. sericata peptidases. The individual clusters encoding for aspartic (Fig1), cysteine (Fig2), metallo (Fig3), threonine (Fig4) and serine (Fig5) peptidases are depicted on the left, while corresponding clans, families and MEROPS IDs are depicted on the right. Shown are log2-transformed RPKM values (blue resembles lower-expressed genes, while red represents highly expressed genes). The supplementary figure 6 shows the melting curve analysis of all genes which were tested via qRT-PCR. [file 8285428.f1.zip › AF3_Cysteine_HM.pdf]

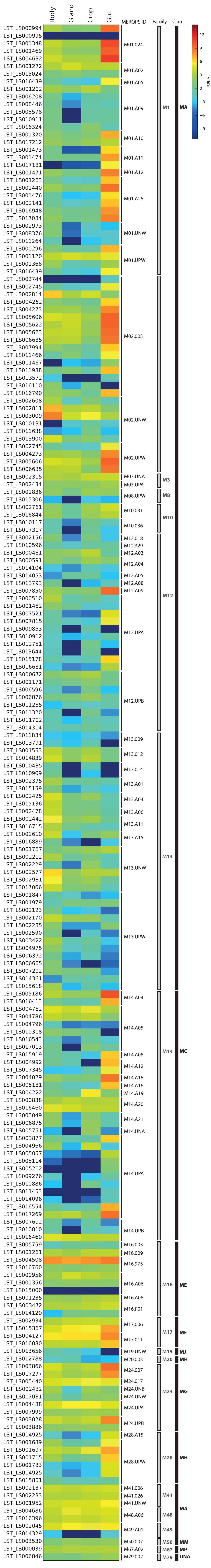

Supplement: Supplementary file 1 — The supplementary table 1 shows number of L. sericata transcripts assembled with Trinity and Oases. K-mer sizes vary between 21 and 75 for the Oases assembly. The supplementary figures 1 to 5 show Heat maps with relative expression levels of all L. sericata peptidases. The individual clusters encoding for aspartic (Fig1), cysteine (Fig2), metallo (Fig3), threonine (Fig4) and serine (Fig5) peptidases are depicted on the left, while corresponding clans, families and MEROPS IDs are depicted on the right. Shown are log2-transformed RPKM values (blue resembles lower-expressed genes, while red represents highly expressed genes). The supplementary figure 6 shows the melting curve analysis of all genes which were tested via qRT-PCR. [file 8285428.f1.zip › AF4_Metallo_HM.pdf]

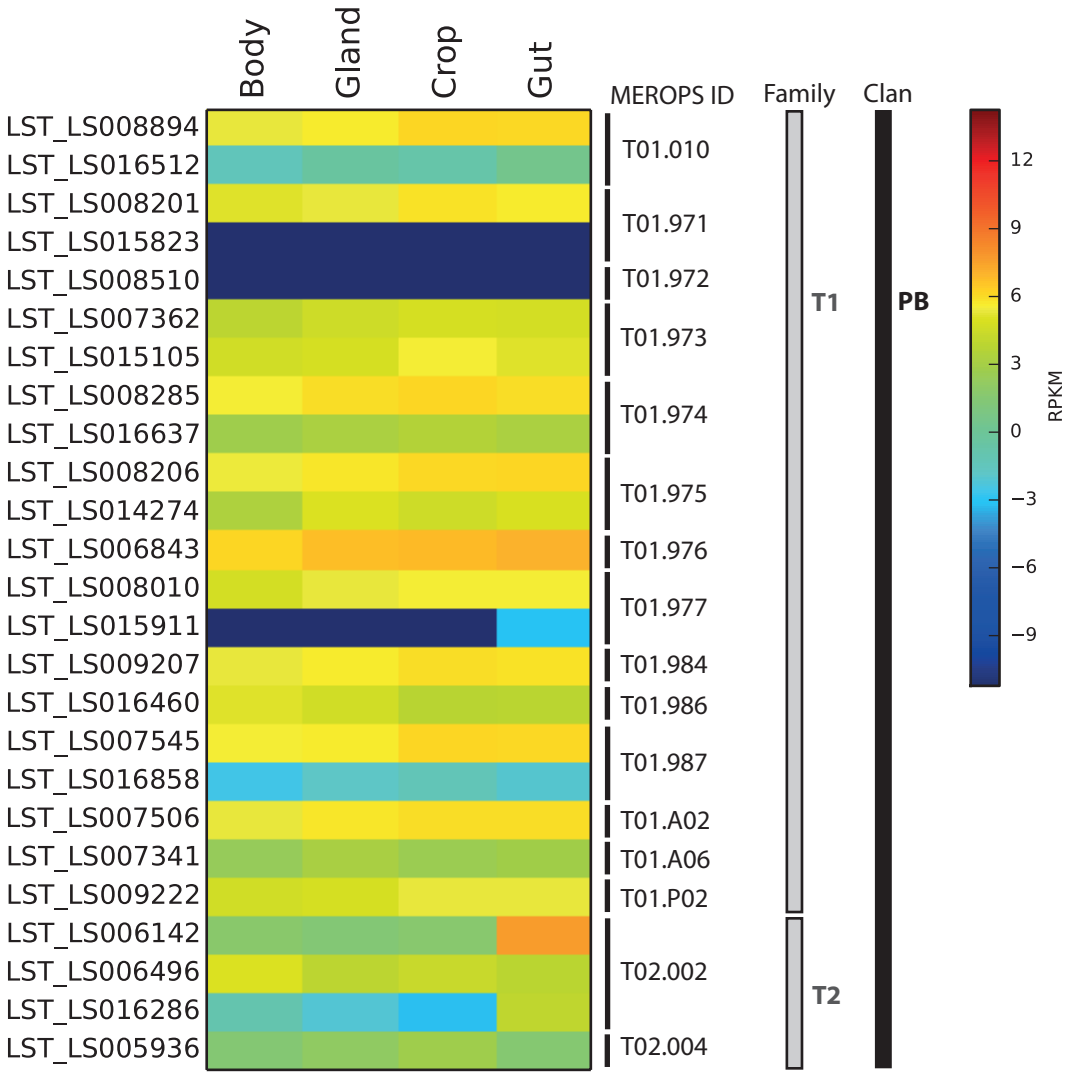

Supplement: Supplementary file 1 — The supplementary table 1 shows number of L. sericata transcripts assembled with Trinity and Oases. K-mer sizes vary between 21 and 75 for the Oases assembly. The supplementary figures 1 to 5 show Heat maps with relative expression levels of all L. sericata peptidases. The individual clusters encoding for aspartic (Fig1), cysteine (Fig2), metallo (Fig3), threonine (Fig4) and serine (Fig5) peptidases are depicted on the left, while corresponding clans, families and MEROPS IDs are depicted on the right. Shown are log2-transformed RPKM values (blue resembles lower-expressed genes, while red represents highly expressed genes). The supplementary figure 6 shows the melting curve analysis of all genes which were tested via qRT-PCR. [file 8285428.f1.zip › AF5_Threonine_HM.pdf]
